# Inhibition of glioblastoma cell proliferation, invasion, and mechanism of action of a novel hydroxamic acid hybrid molecule

**DOI:** 10.1038/s41420-018-0103-0

**Published:** 2018-09-26

**Authors:** Issan Zhang, Maja Beus, Ursula Stochaj, Phuong Uyen Le, Branka Zorc, Zrinka Rajić, Kevin Petrecca, Dusica Maysinger

**Affiliations:** 1 Department of Pharmacology and Therapeutics, McGill University, Montreal, QC Canada; 2 Faculty of Pharmacy and Biochemistry, University of Zagreb, Zagreb, Croatia; 3 Department of Physiology, McGill University, Montreal, QC Canada; 4 Brain Tumour Research Centre, Montreal Neurological Institute and Hospital, Department of Neurology and Neurosurgery, McGill University, Montreal, QC Canada

**Keywords:** Pharmacology, Experimental models of disease, Cancer

## Abstract

Glioblastoma multiforme is one of the most aggressive brain tumors and current therapies with temozolomide or suberoylanilide hydroxamic acid (SAHA, vorinostat) show considerable limitations. SAHA is a histone deacetylase (HDAC) inhibitor that can cause undesirable side effects due to the lack of selectivity. We show here properties of a novel hybrid molecule, sahaquine, which selectively inhibits cytoplasmic HDAC6 at nanomolar concentrations without markedly suppressing class I HDACs. Inhibition of HDAC6 leads to significant α-tubulin acetylation, thereby impairing cytoskeletal organization in glioblastoma cells. The primaquine moiety of sahaquine reduced the activity of P-glycoprotein, which contributes to glioblastoma multiforme drug resistance. We propose the mechanism of action of sahaquine to implicate HDAC6 inhibition together with suppression of epidermal growth factor receptor and downstream kinase activity, which are prominent therapeutic targets in glioblastoma multiforme. Sahaquine significantly reduces the viability and invasiveness of glioblastoma tumoroids, as well as brain tumor stem cells, which are key to tumor survival and recurrence. These effects are augmented with the combination of sahaquine with temozolomide, the natural compound quercetin or buthionine sulfoximine, an inhibitor of glutathione biosynthesis. Thus, a combination of agents disrupting glioblastoma and brain tumor stem cell homeostasis provides an effective anti–cancer intervention.

## Introduction

Glioblastoma multiforme (GBM) is the most common and aggressive form of brain cancer, with limited treatment options and dismal survival rates. Current treatment involves surgical resection followed by radiotherapy and chemotherapy with temozolomide (TMZ)^1^. However, more than half of GBM patients do not respond to TMZ due to the overexpression of DNA repair enzymes, notably *O*^6^-methylguanine transferase^2–4^.

Histone deacetylase (HDAC) inhibitors exert anticancer effects by inducing cell differentiation, cell cycle arrest, and apoptotic cell death through the upregulation of tumor suppressor and cell cycle-regulatory genes^5^. Suberoylanilide hydroxamic acid (SAHA, vorinostat) is a Food and Drug Administration-approved drug for the treatment of cutaneous T cell lymphoma. It is currently in clinical trials for GBM as monotherapy and combined with radiotherapy^6–9^. Despite advancements in treatments, the median survival rate for GBM remains low (14–16 months) and new therapeutic options are urgently needed^3,10^.

In this study, we combined hydroxamic acid—the active moiety of SAHA exerting biological effects in cancer cells—with primaquine to generate a new class of hybrid anticancer agents: sahaquines. Hydroxamic acid inhibits HDACs; these enzymes are overexpressed in many cancers, including GBM^11,12^. The hydroxamic acid pharmacophore of SAHA chelates metal ions, thereby inhibiting metalloenzymes such as HDACs and matrix metalloproteinases (MMPs), which promote cancer growth and invasiveness^13–15^. Hydroxamic acid is a weak acid, which is favorable in the acidic tumor microenvironment as weak bases become protonated, resulting in ion trapping, lysosomal accumulation, elimination by lysosomal exocytosis, and overall decreased biological activity^16–18^. Primaquine can directly interfere with endosomal trafficking to the plasma membrane^19^, inhibit the multidrug resistance transporter P-glycoprotein, and autophagy, thereby sensitizing cancer cells to anti-mitotic drugs^20,21^. Considering that monotherapies have limited effectiveness in GBM, we tested sahaquine in combination with TMZ, the standard of care for GBM, quercetin, and buthionine sulfoxamine. Quercetin is an abundant flavonoid found in fruits and vegetables, such as apples and onions. Its estimated daily intake ranges from 3–40 mg, but supplements up to 1000 mg per day are considered safe^22^. Although it shows no toxicity in normal cells, several studies have shown that quercetin has anticancer effects. Its mechanism of action involves the upregulation of pro-apoptotic and downregulation of anti-apoptotic factors, cell cycle arrest, and DNA intercalation, resulting in DNA damage, activation of apoptosis, and cell death^23^. In animal studies, quercetin inhibited tumor growth and improved the lifespan of tumor-bearing mice^23,24^. Furthermore, the anticancer effects of quercetin are enhanced in combination with chemotherapeutic agents or other drugs^25–27^.

We investigated the loss of cell viability and invasiveness in GBM as functional read-outs of the effects of sahaquine alone or in combination with TMZ and quercetin. Sahaquine was tested in both differentiated GBM cells and brain tumor stem cells (BTSCs), which are key to tumor survival and recurrence^28–31^. Our study supports the model that sahaquine-induced cell death of GBM is mediated through multiple pathways, including inhibition of HDAC6, reduction of epidermal growth factor receptor (EGFR) protein abundance, and decreased activation of downstream kinases AKT and ERK1/2. The primaquine moiety of sahaquine contributes to the inhibition of P-glycoprotein. Considering that sahaquine significantly reduced BTSC viability and markedly inhibited GBM invasion by disruption of GBM homeostasis, further systematic studies are warranted in patient-derived organoids.

## Results

### Sahaquine synthesis and physicochemical properties of the selected anticancer agents

**Figure 1:**
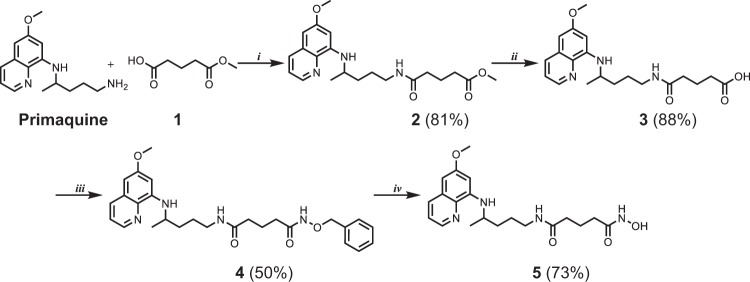
Synthesis of sahaquine and its precursors. Reagents and conditions: (i) HATU, DIEA, dichloromethane, 1 h, (ii) LiOH, methanol, H_2_O, 1 h, (iii) *O*-benzylhydroxylamine, HATU, DIEA, dichloromethane, 2 h, (iv) H_2_, 10% Pd/C, methanol, 4 h. All reactions were performed at room temperature. Yields are shown in brackets. HATU 1-[*bis*(dimethylamino)methylene]-1*H*-1,2,3-triazolo[4,5-*b*]pyridinium 3-oxid hexafluorophosphate, DIEA *N,N*-diisopropylethylamine, LiOH lithium hydroxide)

Sahaquine is a primaquine and hydroxamic acid derivative linked with glutaric acid. It is synthesized in four steps (Fig. 1). The pharmacophore, hydroxamic acid, was introduced in the last step. Yields were good to excellent (50–88%). Sahaquine was fully characterized by conventional spectroscopic and analytical methods (melting point, IR, MS, ^1^H-NMR, ^13^C-NMR), and the data were consistent with the proposed structure (Supplementary Fig. S1). The quinoline ring of sahaquine acts as the capping group and the hydroxamic acid binds zinc. Calculations of physicochemical properties showed that TMZ is a hydrophilic compound (log *P* = –0.28), whereas sahaquine and particularly quercetin are more lipophilic (log *P* = 0.92 and 2.16, respectively) (Table 1). The isoelectric point (pI) values of these compounds vary from 2.9 (quercetin) to 9.2 (SAHA)^32^.

**Table 1:** Structures of sahaquine, temozolomide, quercetin, and SAHA with basic physicochemical properties

The physicochemical properties are calculated with the Chemicalize.org program (Instant Cheminformatics Solutions. Available online at http://www.chemicalize.org/ (accessed on 10 October 2017))

### Sahaquine is more potent than TMZ for killing human glioblastoma and BTSCs

**Figure 2:**
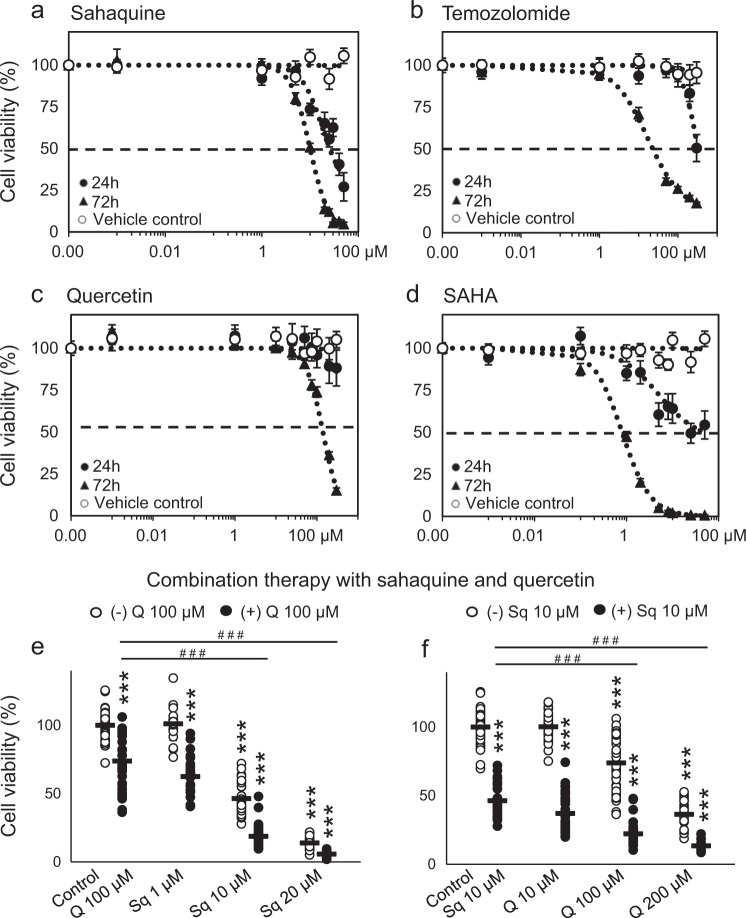
Sahaquine kills human glioblastoma cells in a dose-dependent and time-dependent manner. GBM cell viability (*n* = 9) following treatment with **a** sahaquine (Sq, 0.001–50 µM), **b** temozolomide (TMZ, 0.001–500 µM), **c** quercetin (Q, 0.001–300 µM), and **d** SAHA (0.001–50 µM) for 24 or 72 h. Shown is the mean (SEM) percentage cell viability compared to untreated controls from three independent experiments. **e**, **f** Dose-dependent decrease in cell viability (72 h) with the combination of either a fixed concentration of quercetin (100 µM, n = 41) and increasing concentrations of sahaquine (Sq 1 µM, *n* = 21, Sq 10 µM, *n* = 30, Sq 20 µM, *n* = 21, Sq 1 µM + Q, *n* = 25, Sq 10 µM + Q, *n* = 29, Sq 20 µM + Q, *n* = 27), or a fixed concentration of sahaquine (10 µM *n* = 30) and increasing concentrations of quercetin (Q 10 µM, *n* = 37, Q 100 µM, *n* = 41, Q 200 µM, *n* = 28, Q 10 µM + Sq, *n* = 34, Q 100 µM + Sq, *n* = 35, Q 200 µM + Sq, *n* = 21). Each point represents a percentage value normalized to the untreated control (**e** *n* = 47, **f** *n* = 125). Horizontal bars represent the mean (SD) from at least three independent experiments (***p < 0.001 compared to the untreated control; ^###^*p* < 0.001 compared to **e** Q 100 µM alone or **f** Sq 10 µM alone; Welch’s ANOVA with Games–Howell post hoc test). Cell viability was measured by counting Hoechst 33342-labeled nuclei imaged using a fluorescence microscope

The half maximal inhibitory concentration (IC_50_) value of sahaquine (10 µM) was about threefold lower than that of TMZ (31 µM), whereas it was less potent than its parent compound SAHA after 72 h incubation (Fig. 2). Sahaquine precursors were also tested, but because of the relatively high IC_50_ values (>50 µM), further experiments were not pursued (Supplementary Table S1). Enhanced cell killing was achieved by combining quercetin with sahaquine in a dose-dependent manner, although quercetin alone showed limited cytotoxicity (IC_50_ = 140 µM after 72 h) (Fig. 2e). Combination of TMZ with sahaquine, quercetin, or SAHA at IC_50_ concentrations was more effective than any of the compounds alone (Supplementary Fig. S2). Similar results were obtained by measurements of mitochondrial metabolic activity using the MTT (3-[4,5-dimethylthiazol-2-yl]-2,5-diphenyl tetrazolium bromide) assay (Supplementary Fig. S3).

We further tested the selected compounds on GBM tumoroids, which are more drug-resistant and representative models of brain tumors in vivo. Sahaquine and TMZ reduced tumoroid sizes by 37 and 40%, respectively, while quercetin did not have a significant effect after 7 days (Supplementary Fig. S4).

**Figure 3:**
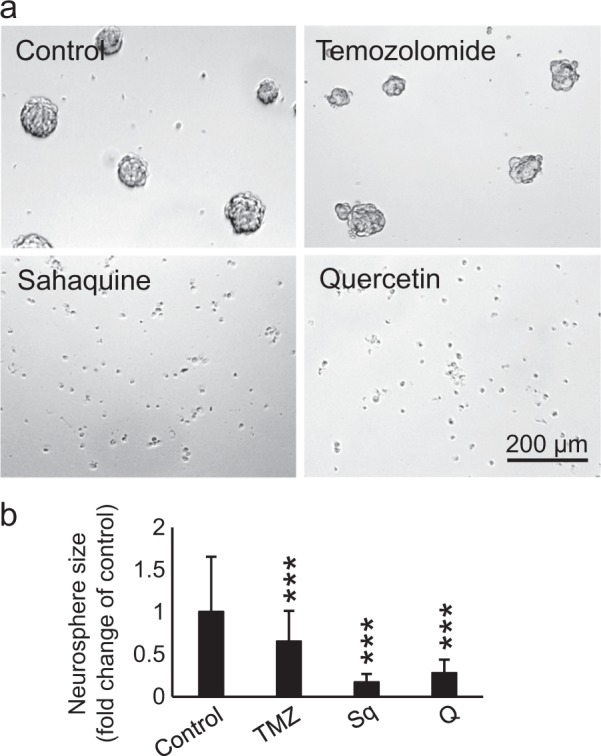
Sahaquine and quercetin are most effective in killing human brain cancer stem cells. **a** Representative micrographs of BTSCs treated with temozolomide (100 μM, *n* = 99), sahaquine (10 μM, *n* = 183) or quercetin (100 μM, *n* = 264) for 7 days. **b** The surface area of neurospheres was measured based on 2D micrographs from three independent experiments. Shown are average values (SD) normalized to the untreated controls (set to 1, *n* = 334) (***p < 0.001, Welch’s ANOVA with Games–Howell post hoc test)

Based on the results shown in Fig. 2, we investigated the cytotoxic effects of the selected compounds on BTSCs. BTSCs are a key subpopulation of GBM tumors implicated in tumor initiation, propagation and recurrence^28,30^. In vitro BTSC cultures spontaneously formed neurospheres of approximately 100 µm in diameter within 7 days. Sahaquine and quercetin were most effective at reducing the size of BTSC aggregates and abolishing the formation of neurospheres (Fig. 3).

### Sahaquine inhibits GBM invasion and P-glycoprotein activity

**Figure 4:**
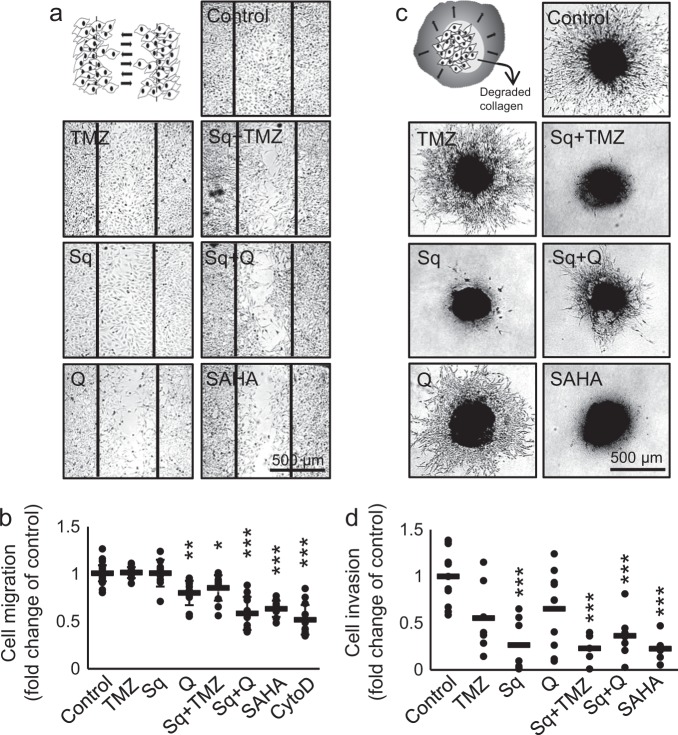
Sahaquine inhibits human glioblastoma cell migration and invasion. Cell migration was measured using the scratch assay, as schematically represented in **a**. Representative micrographs show migration of cells into the scratch (delineated by vertical black bars) after treatment with temozolomide (100 µM, *n* = 12), sahaquine (Sq, 10 µM, *n* = 10), quercetin (Q, 100 µM, *n* = 12) or SAHA (10 µM, *n* = 10), alone or in combination (Sq + TMZ, *n* = 12; Sq + Q, n = 12) for 24 h. Cytochalasin D (40 nM, *n* = 13) served as a positive control. **b** Cell migration was quantified as the area covered by migrating cells. Each point represents a value normalized to the untreated control (set to 1, *n* = 35). Horizontal bars represent the mean (SD) from at least three independent experiments (*p < 0.05, **p < 0.01, ***p < 0.001, Welch’s ANOVA with Games–Howell post hoc test). **c** Cell invasion was measured from the radial movement of cells from 3D tumoroids embedded in a collagen matrix, as schematically represented in the first panel. Representative micrographs show cell movement from 3D tumoroids treated with temozolomide (100 µM, *n* = 7), sahaquine (Sq, 10 µM, *n* = 7), quercetin (Q, 100 µM, *n* = 10), or SAHA (10 µM, *n* = 5), alone or in combination (Sq + TMZ, *n* = 5; Sq + Q, *n* = 8) after 4 days. **d** Cell invasion was quantified from the area covered by invading cells. Each point represents a value normalized to the untreated control (set to 1, *n* = 11). Horizontal bars represent the mean from at least three independent experiments (***p < 0.001, two-tailed one-way ANOVA with Tukey–Kramer’s post hoc test)

GBM is characterized by a diffuse brain tissue distribution^33^. Tumors commonly reoccur within a few centimeters of the original lesion, making surgical resection difficult^33^. We tested the effect of sahaquine and quercetin on GBM migration using a scratch assay, and invasion using a three-dimensional (3D) collagen matrix. Sahaquine did not significantly inhibit cell migration over 24 h, while quercetin reduced cell migration by 20% (Fig. 4a). The combination of sahaquine with quercetin was most effective, reducing migration by 42%. This effect was not observed when combining sahaquine with TMZ. In contrast, sahaquine significantly inhibited GBM invasiveness, whereas quercetin and TMZ reduced cell invasion by 35 and 45% after 4 days, respectively (Fig. 4c). GBM invasiveness is enabled by MMP degradation of the extracellular matrix and basement membranes^34,35^. We investigated the effect of the selected compounds on the abundance of secreted MMPs using gelatin zymography and showed that quercetin decreases MMP abundance in a dose-dependent manner (Supplementary Fig. S5). Neither sahaquine nor TMZ reduced MMP concentrations, although the hydroxamic acid moiety in sahaquine can bind zinc within the MMP structure^36^. The primaquine moiety of sahaquine contributed to the inhibition of P-glycoprotein, as assessed by intracellular retention of calcein-AM (Supplementary Fig. S6). The primaquine concentration within sahaquine (10 µM) effectively inhibited P-glycoprotein activity, whereas 60 µM of unincorporated primaquine was required to achieve a comparable effect. A smaller extent of P-glycoprotein inhibition by SAHA was obtained with equimolar sahaquine concentrations (10 µM) (Supplementary Fig. S6).

### Sahaquine selectively inhibits HDAC6

**Figure 5:**
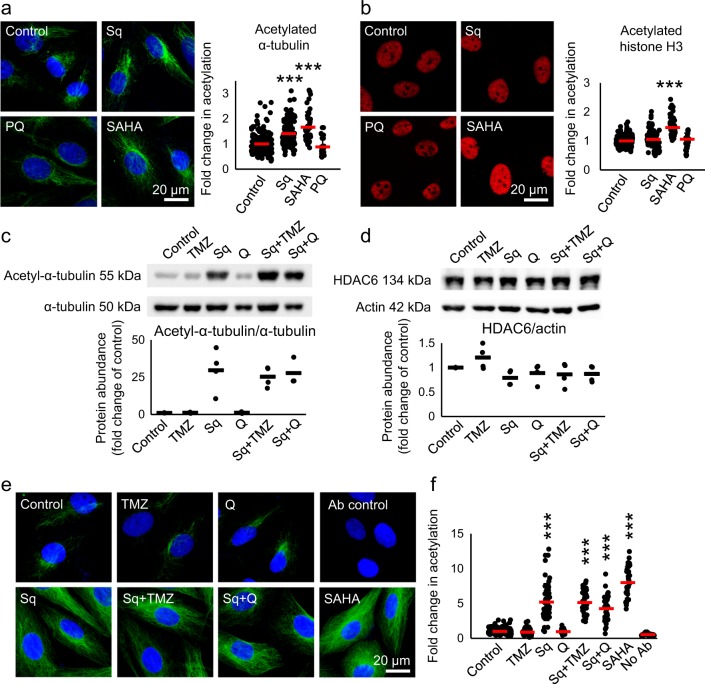
Sahaquine-mediated HDAC6 inhibition results in selective α-tubulin hyperacetylation at nanomolar concentrations. **a** Representative fluorescence micrographs of GBM α-tubulin acetylation (green) at lysine 40 in response to sahaquine (Sq, 100 nM, *n* = 121 cells), SAHA (100 nM, *n* = 54 cells) or primaquine (PQ, 10 µM, *n* = 30 cells) treatment for 24 h. **b** Representative fluorescence micrographs of GBM histone H3 acetylation (red) at lysine 9/lysine 14 in response to sahaquine (Sq, 100 nM, *n* = 120 cells), SAHA (100 nM, *n* = 90 cells) or primaquine (PQ, 10 µM, *n* = 83 cells) for 24 h. Nuclei (blue) were labeled with Hoechst 33342. Cells were imaged using a fluorescence microscope and fluorescence was analyzed in ImageJ. Shown are averages of fluorescence per cell (SD) normalized to the untreated controls (set to 1) from at least three independent experiments (***p < 0.001, Welch’s ANOVA with Games–Howell post hoc test). **c** Acetylated α-tubulin (*n* = 3) and **d** HDAC6 protein abundance (*n* = 4) in GBM cells treated with temozolomide (TMZ, 100 µM), sahaquine (Sq, 10 µM) or quercetin (Q, 100 µM) alone or in combination for 24 h, measured by Western blotting. Acetylated α-tubulin and HDAC6 were normalized to total α-tubulin and actin, respectively. Each point represents a value normalized to the untreated control (set to 1). Horizontal bars represent the mean from at least three independent experiments. **e** Representative fluorescence micrographs of GBM α-tubulin acetylation (green) at lysine 40 in response to temozolomide (TMZ, 100 µM, *n* = 76 cells), sahaquine (Sq, 10 µM, *n* = 56 cells), quercetin (Q, 100 µM, *n* = 32 cells) or SAHA (10 µM, *n* = 36 cells) alone or in combination for 24 h. Nuclei (blue) were labeled with Hoechst 33342. **f** Shown are averages (SD) of fluorescence per cell normalized to the untreated control (set to 1, *n* = 197 cells) from at least three independent experiments (***p < 0.001, Welch’s ANOVA with Games–Howell post hoc test)

We further examined the HDAC inhibitory activity of sahaquine compared to its parent compound SAHA. SAHA is a pan-HDAC inhibitor that caused an increase in both acetylated α-tubulin (K40) and acetylated histone H3 (K9/K14) (Fig. 5). We hypothesized sahaquine to be selective toward HDAC6, because its bulky capping group would fit better into the wide binding site of the enzyme^37,38^. Nanomolar concentrations (100 nM) of sahaquine resulted in a 1.5-fold increase in acetylated α-tubulin compared to the untreated control, but did not affect histone acetylation (Fig. 5a). Similar results were obtained with the HDAC6-selective inhibitor ACY-1215 (Supplementary Fig. S7). TMZ and quercetin did not inhibit HDAC6. These results were supported by Western blot analyses (Fig. 5c). HDAC6 abundance was comparable following all treatments, suggesting that sahaquine inhibited the enzyme activity without affecting its protein levels (Fig. 5d).

### Sahaquine reduces EGFR abundance, ERK1/2, and AKT phosphorylation

**Figure 6:**
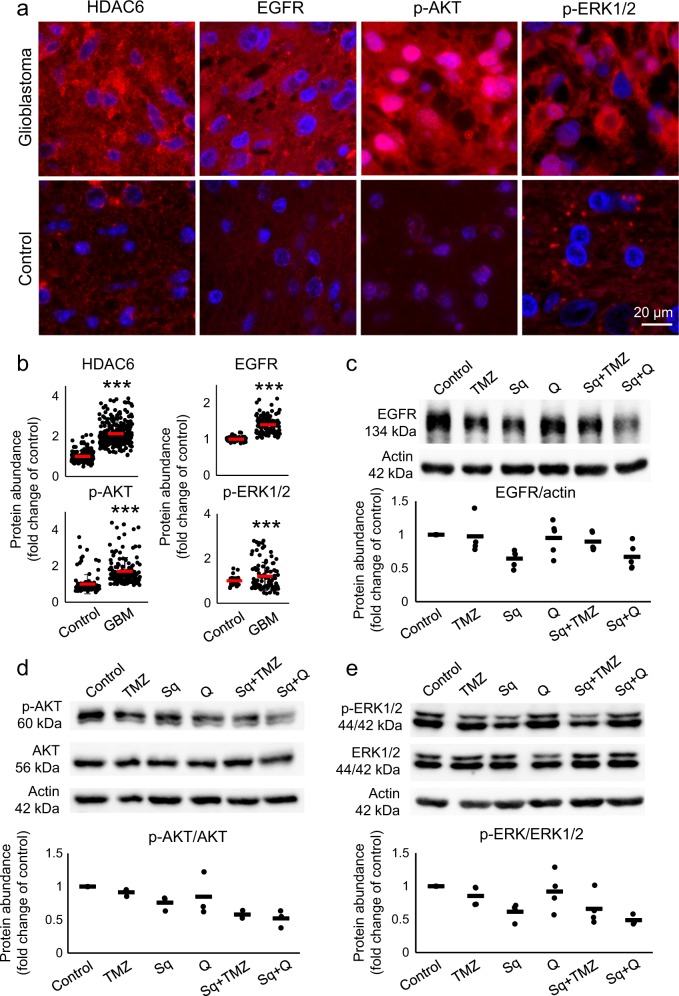
Sahaquine reduces EGFR abundance and AKT/ERK1/2 phosphorylation in human glioblastoma. **a** Representative fluorescence micrographs of human brain sections (GBM or healthy control) labeled for HDAC6, EGFR, phosphorylated AKT (p-AKT), or dually phosphorylated ERK1/2 (p-ERK1/2). Nuclei (blue) were labeled with Hoechst 33342. Cells were imaged using a fluorescence microscope and fluorescence was analyzed in ImageJ. **b** Horizontal bars represent averages of fluorescence per cell (SD) for HDAC6 (control, *n* = 181 cells, GBM, *n* = 272 cells), EGFR (control, *n* = 94 cells, GBM, *n* = 116 cells), p-AKT (control, *n* = 115 cells, GBM, *n* = 147 cells) and p-ERK1/2 (control, *n* = 35 cells, GBM, *n* = 104 cells). Each point represents a value normalized to the healthy control (set to 1) (***p < 0.001, Welch’s ANOVA with Games–Howell post hoc test). **c** EGFR (TMZ, *n* = 4, Sq, *n* = 5, Q, *n* = 5, Sq + TMZ, *n* = 5, Sq + Q, *n* = 5), **d** phosphorylated AKT (*n* = 3), and **e** phosphorylated ERK1/2 (TMZ, *n* = 4, Sq, *n* = 4, Q, *n* = 4, Sq + TMZ, *n* = 4, Sq + Q, *n* = 3) protein abundances were measured in GBM cells treated with temozolomide (TMZ, 100 µM), sahaquine (Sq, 10 µM), or quercetin (Q, 100 µM) alone or in combination for 24 h, by Western blotting. EGFR protein abundance was normalized to the actin loading control. Phosphorylated AKT and ERK1/2 were normalized to total AKT and total ERK1/2, respectively. Each point represents a value normalized to the untreated control (set to 1). Horizontal bars represent means from at least three independent experiments

EGFR overexpression and downstream hyperactivity of ERK1/2 and AKT are associated with worse prognosis in GBM^39,40^. We assessed the abundance of these markers and HDAC6 in GBM by immunohistochemistry, and showed an increase in EGFR, dually phosphorylated (active) ERK1/2, phosphorylated (active) AKT, and HDAC6 compared to control brains (Fig. 6a). To test whether sahaquine impinges on EGFR and the activation of downstream kinases, we measured EGFR abundance, dual ERK1/2 phosphorylation (Thr202/Tyr204), and AKT phosphorylation (Ser473) by Western blotting (Fig. 6b). Sahaquine reduced EGFR concentrations in GBM. Interestingly, combining sahaquine and TMZ abrogated this inhibitory effect. Sahaquine also reduced levels of phosphorylated ERK1/2 and phosphorylated AKT, alone and in combination with quercetin or TMZ. Total ERK1/2 and AKT protein levels remained unchanged (Supplementary Fig. S8).

## Discussion

Results from this study show that sahaquine is more effective than TMZ in killing glioblastoma and BTSCs, as well as inhibiting glioblastoma invasiveness. The mode of action of sahaquine implicates in part excessive α-tubulin acetylation due to the selective inhibition of HDAC6, resulting in cytoskeletal reorganization (Supplementary Fig. S9) and reduced invasiveness. Additional modes of action involve decreased EGFR abundance and downstream activity of AKT and ERK1/2. These results are particularly striking in combination with TMZ or quercetin.

TMZ is one of few clinically approved drugs for the treatment of GBM, but a substantial portion of newly diagnosed tumors and recurrent tumors are resistant to this drug^3,41^. HDAC inhibitors are of particular interest for GBM treatment, as their effectiveness is unaltered by mechanisms of resistance upregulated in GBM, such as mismatch-repair, *O*^6^-methylguanine methyltransferase and base-excision repair^3,4,42^. The pan-HDAC inhibitor SAHA is currently in clinical trials for GBM, but results so far showed marginal improvement in overall survival (5.7 months compared to 4.4 months) and several serious side effects^6–9,43,44^. This and other current therapeutic interventions for GBM are ineffective^28,29,31^.

Thus, our goal was to test a new hybrid compound. The development of hybrid molecules is one of the most active areas in therapeutics. Hybrid compounds can have multiple targets, reducing the risk of resistance, lowering effective doses, and decreasing side effects^45,46^. Sahaquine is a hybrid molecule consisting of hydroxamic acid and primaquine linked by a dicarboxylic acid. Primaquine is a strong base (pI = 13.7), but addition of the hydroxamic acid group lowers its pI to 6.48, making sahaquine a weak acid. Weak acids are more advantageous than weak bases as anticancer therapeutics, because they will not be protonated in the acidic tumor environment or trigger lysosomal exocytosis^16–18^. Similarly to primaquine, sahaquine can also inhibit P-glycoprotein activity (Supplementary Fig. S6), thereby preventing multidrug resistance.

One of the great challenges in GBM treatment is heterogeneity, both within and between tumors^47–50^. Interpatient heterogeneity has been shown through genomic and transcriptomic analyses by the Cancer Genome Atlas research network^51,52^. Intratumoral heterogeneity can be attributed to the different cellular lineages and subtypes present in different parts of the same tumor^53,54^, or even in individual cells within a tumor^52,55^. Another cause of GBM heterogeneity is the presence of BTSCs, a subset of glioma cells with the abilities of self-renewal, differentiation, and recapitulation of the original tumor upon xenotransplantation^56–58^. They are resistant to radiation^28^ and chemotherapy^59–61^, and are thought to promote tumor recurrence^30,31^. Therefore, effective GBM treatment demands a better understanding of tumor origin and heterogeneity to identify new therapeutic targets^3,62^. Sahaquine (10 µM) abolished the formation of BTSC neurospheres and significantly reduced the size of BTSC aggregates. TMZ was less effective, even at a tenfold higher concentration (100 µM). Quercetin was as effective as sahaquine in killing BTSCs, but showed limited cytotoxicity toward differentiated GBM cells. Sahaquine eliminated both BTSCs and differentiated cancer cells.

Another factor contributing considerably to GBM recurrence is tumor invasiveness. While sahaquine abolished invasiveness and contributed to the loss of tumoroid viability, it did not markedly affect the abundance of secreted MMPs. In contrast, quercetin had limited effects on tumoroid viability, but decreased GBM invasion by inhibiting MMP secretion. Quercetin inhibits nuclear factor-κB (NF-κB) nuclear translocation, which could alter MMP expression^63,64^ and enhance cell death through NF-κB-dependent regulation of apoptosis.

In an effort to reduce undesirable side effects in normal cells, selective HDAC inhibitors have been developed^65,66^. Ricolinostat (ACY-1215) is a selective HDAC6 inhibitor currently in clinical trials (phase I and II) in combination with pomalidomide for multiple myeloma^67^. Ricolinostat inhibits heat shock protein 90 deacetylation, resulting in an accumulation of unfolded proteins, disruption of protein homeostasis and cell death^68^. We show that sahaquine selectively inhibits HDAC6 at nanomolar concentrations, which distinguishes it from SAHA, which is non-selective at equimolar concentrations. Interestingly, sahaquine significantly reduced the abundance of heat shock protein 70 in GBM (Supplementary Fig. S10) and altered α-tubulin organization. We have previously shown that celastrol disrupts protein homeostasis^69^ and the organization of the F-actin cytoskeleton in GBM^70^. Future studies will have to evaluate how sahaquine affects proteostasis in relation to cytoskeletal dynamics.

Many drugs currently in clinical trials aim at inhibiting proteins and proliferation pathways deregulated in GBM, notably HDAC6, EGFR, AKT, and ERK1/2^71–74^. Our in vitro studies showing enhanced ERK1/2 and AKT phosphorylation are corroborated by immunohistochemical data in tumor sections from GBM patients (Fig. 6a), also showing markedly stronger signals for HDAC6, EGFR, phosphorylated ERK1/2, and phosphorylated AKT compared to normal brain tissue (Fig. 6a). Sahaquine can decrease the abundance of EGFR, phosphorylated AKT, and phosphorylated ERK1/2 in GBM (Fig. 6b), thereby suggesting that similar hybrid molecules are viable candidates for GBM combination therapy. Interestingly, AKT deacetylation by HDAC6 promotes cancer growth and proliferation^75^, indicating that sahaquine could reduce AKT activation through HDAC6 inhibition.

Drug resistance is a major problem in glioblastoma therapy^3,41^. A recent study of HDAC inhibitors in drug-resistant melanoma implicated increased levels of reactive oxygen species^76^. Combination of sahaquine with buthionine sulfoximine, which depletes endogenous glutathione levels^77^, sensitized GBM cells to reactive oxygen species and enhanced cell death (Supplementary Fig. S11), although buthionine sulfoximine alone in the tested concentration had no effect on GBM viability. Further analysis of the effect of sahaquine on reactive oxygen species production in GBM is warranted.

**Figure 7:**
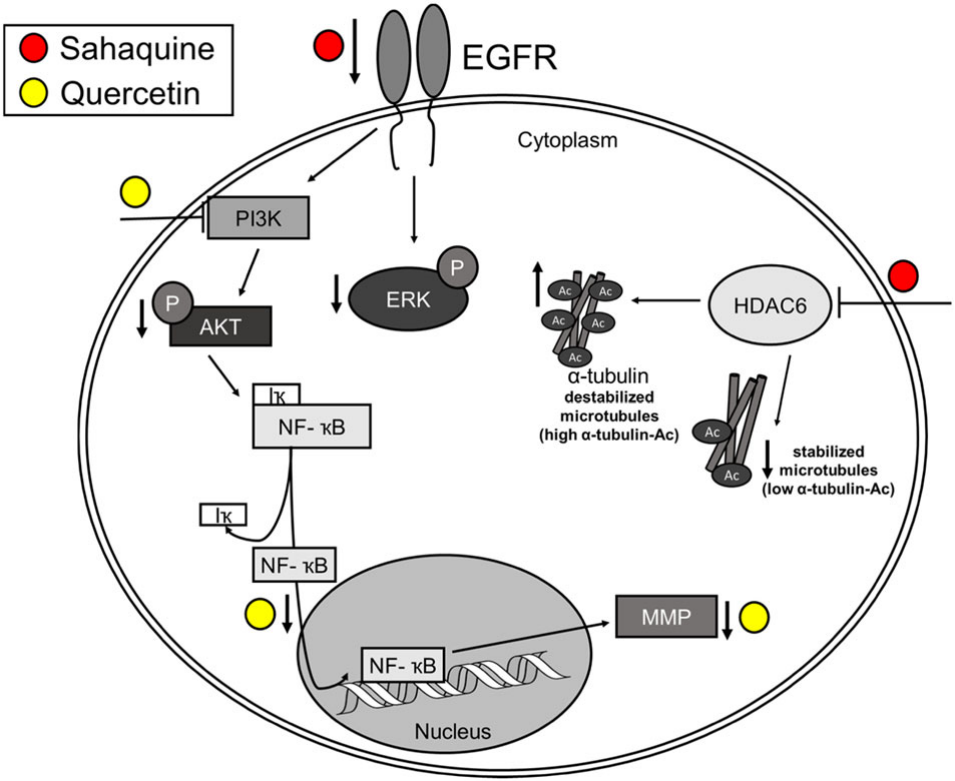
Proposed mechanism of action of sahaquine and quercetin in GBM

Taken together, our study reveals sahaquine as a therapeutic agent affecting multiple cellular factors and processes that are critical for GBM treatment (Fig. 7). Sahaquine is superior to the clinical standard TMZ in reducing GBM and BTSC viability, invasiveness, and markers of key survival pathways. These effects are even more profound when sahaquine is combined with TMZ, buthionine sulfoximine, or quercetin. In conclusion, sahaquine is an effective cell death inducer which eliminates not only GBM cells but also BTSCs, thus suggesting that evaluation of sahaquine in combination with other drugs merit further investigations in patient-derived organoids, and eventually in humans.

## Materials and methods

### Synthesis of sahaquine

Sahaquine **5** was synthesized in four reaction steps (Fig. 1) adapted from the synthetic approach of Zhang et al^45^. Details of these steps are provided in the Supplementary Information. The first step included amide bond formation between mono-methyl glutarate (**1**, Sigma-Aldrich, St. Louis, MO, USA) and primaquine (Sigma-Aldrich, St. Louis, MO, USA), with 1-[*bis*(dimethylamino)methylene]-1*H*-1,2,3-triazolo[4,5-*b*]pyridinium 3-oxid hexafluorophosphate (HATU, Alfa Aesar, Thermo Fisher, Kandel, Germany) as a coupling agent and *N,N*-diisopropylethylamine (DIEA, Alfa Aesar, Thermo Fisher, Kandel, Germany) as a base. The prepared product **2** was further hydrolyzed with lithium hydroxide (Sigma-Aldrich, St. Louis, MO, USA) and gave carboxylic acid **3**. In the next step, **3** was coupled with *O*-benzylhydroxylamine (Sigma-Aldrich, St. Louis, MO, USA) in the presence of HATU/DIEA and yielded *O*-benzylhydroxamic acid **4**, which was deprotected by catalytic hydrogenation and gave the target compound **5** (sahaquine). All reactions proceeded at room temperature.

### Cell culture and tissue samples

U251N human glioblastoma cells were originally obtained from the American Type Culture Collection. Cells were cultured in Dulbecco’s modified Eagle's media (DMEM, Gibco, Thermo Fischer Scientific, Grand Island, NY, USA) supplemented with 5% (v/v) fetal bovine serum (Wisent, St. Bruno, Canada) and 1% (v/v) penicillin–streptomycin (Thermo Fisher Scientific, Eugene, OR, USA) at 37 °C with 5% CO_2_ and 95% relative humidity, unless otherwise indicated. Glioblastoma samples were harvested under a protocol approved by the Montreal Neurological Hospital’s research ethics board (NEU-10-066). Consent was given by all patients. At least 116 brain sections from GBM patients aged 55–76 and controls were used. Tissues were from the frontal, temporal, or parietal lobes of the cerebral cortex. Human BTSCs were expanded as neurospheres in complete NeuroCult^TM^ proliferation media (Stemcell Technologies, Vancouver, BC, Canada). NeuroCult^TM^ basal medium contained: NeuroCult^TM^ NS-A proliferation supplement (1/10), recombinant human epidermal growth factor EGF (20 ng/ml), recombinant human basic fibroblast growth factor (20 ng/ml), and heparin (2 μg/ml).

### Cell counting assay

U251N cells were seeded in 96-well black plates (Costar, Corning, NY, USA) at 5,000 cells per well in 0.1 ml media and cultured for 24 h. Cells were treated with sahaquine (0.001, 1, 5, 10, 20, 25, 30, 40, and 50 µM), TMZ (0.001, 1, 10, 50, 100, 200, 300, 400, and 500 µM, Sigma-Aldrich, St. Louis, MO, USA), quercetin (0.001, 1, 10, 25, 50, 75, 100, 200, and 300 µM, Sigma-Aldrich, St. Louis, MO, USA), or SAHA (0.001, 0.1, 1, 2, 5, 8, 10, 25, and 50 µM, Cayman Chemical, Ann Arbor, MI, USA) for 24 or 72 h. Combination treatments included increasing concentrations of sahaquine (0.001, 1, 3, 5, 7, 10, 20, and 50 µM) with quercetin (100 µM), increasing concentrations of quercetin (0.001, 1, 10, 25, 50, 100, and 200 µM) with sahaquine (10 µM), TMZ (30 µM) with sahaquine (10 µM), quercetin (140 µM) or SAHA (1 µM), and buthionine sulfoximine (100 µM, Sigma-Aldrich, St. Louis, MO, USA) with sahaquine (10 µM) for 24 h or 72 h. Following treatment, cells were fixed with 4% paraformaldehyde (w/v, 10 min, BDH, Toronto, ON, Canada). Nuclei were labeled with Hoechst 33342 (10 µM, 10 min, Thermo Fisher Scientific, Eugene, OR, USA). Cells were washed with phosphate-buffered saline and imaged using a fluorescence microscope (Leica DMI4000B, Toronto, ON, Canada).

### BTSC viability

48EF human brain tumor cells were seeded at 5,000 cells per well in 96-well plates (Sarstedt, Nümbrecht, Germany) and treated for 7 days. Cells were then imaged using light microscopy (Leica DMI4000B) and the surface areas of the neurospheres were measured in ImageJ (version 1.51s).

### Scratch assay

U251N cells were seeded in 6-well plates (Sarstedt, Nümbrecht, Germany) at 1,500,000 cells per well in 1 ml media and cultured for 24 h. The scratch was performed by gently dragging a 200 μl pipette tip across the cell monolayer, after which cells were washed with phosphate-buffered saline and incubated in DMEM with or without treatment. Cytochalasin D (40 nM, Sigma-Aldrich, St. Louis, MO, USA) served as positive control. Predetermined areas of the wells were imaged using light microscopy immediately after the scratch (time = 0 h) and after 24 h. The cell-free area of the scratch was measured in ImageJ.

### Cell invasion assay

U251N tumoroids were prepared using the hanging drop method^78^. Drops of 30,000 cells in 20 µl medium were pipetted onto the inner side of a 10 cm Petri dish (Thermo Fisher Scientific, Eugene, OR, USA) lid. The lid was quickly flipped to cover the Petri dish filled with 20 ml phosphate-buffered saline. Hanging drops were cultured at 37 °C for 48 h to allow tumoroids to form. Tumoroids were then gently scooped into a medium-filled Petri dish coated with 2% agarose and cultured for 48 h. Tumoroids were implanted in collagen gel (Advanced BioMatrix, San Diego, CA, USA) mixed with DMEM (1×) and sodium hydroxide (10 mM, Sigma-Aldrich, St. Louis, MO, USA). Gels were covered with 500 µl DMEM with or without treatment. Tumoroids were imaged using light microscopy immediately after implantation (time = 0 day) and after 4 days. The area of cell invasion was measured in ImageJ.

### Immunocytochemistry

Following treatment, U251N human glioblastoma cells were fixed with 4% paraformaldehyde (10 min), and then permeabilized using 0.1% Triton X-100 (v/v, 10 min, Sigma-Aldrich, St. Louis, MO, USA). Blocking was performed with 10% goat serum (v/v, 1 h, Thermo Fisher Scientific, Eugene, OR, USA) in phosphate-buffered saline, and then samples were incubated with primary antibodies (acetyl-histone H3 K9/K14, 1/500, Cell Signalling, #9677; acetyl-α-tubulin K40, 1/500, Santa Cruz, sc-23950; α-tubulin, 1/1000, Abcam, ab7291) overnight at 4 °C in a humidified chamber. Samples were washed three times with phosphate-buffered saline with 5 min incubation between washes. Secondary antibodies (anti-rabbit Alexa Fluor 488, 1/500, Thermo Fisher Scientific, A11008; anti-mouse Alexa Fluor 647, 1/500, Thermo Fisher Scientific, A28181) were incubated with samples for 1 h in the dark, and then washed off three times with phosphate-buffered saline with 5 min incubation in between washes. Nuclei were labeled with Hoechst 33342 (10 μM, 10 min). Samples were mounted on microscope slides using Aqua-Poly/Mount (Polysciences, Warrington, PA, USA) and dried overnight before imaging with a fluorescence microscope (Leica DMI4000B).

### Immunohistochemistry

Human brain sections were dewaxed in xylene, and then rehydrated in ethanol. Antigen retrieval was performed in citrate buffer using a decloaking chamber for 3 h. Samples were washed twice with double-distilled water, three times with phosphate-buffered saline, and then blocked with Protein Block (10 min, Spring Biosciences, Pleasanton, CA, USA). Samples were incubated with primary antibodies (HDAC6, 1/100, Santa Cruz, sc-11420; EGFR, 1/100, Oncogene Science Ab-1; phospho-AKT Ser473, Cell Signalling, #9271; phospho-p44/42 Erk1/2 Thr202/Tyr204, 1/100, Cell Signalling, #9101) overnight at 4 °C in a humidified chamber. Samples were washed twice with IF buffer (0.05% (v/v) Tween 20, 0.2% (v/v) Triton X-100 in phosphate-buffered saline). Secondary antibodies (anti-rabbit Alexa Fluor 647, 1/500, Thermo Fisher Scientific, A21245; anti-rabbit Alexa Fluor 488, 1/1000, Thermo Fisher Scientific, A27034; anti-mouse Alexa Fluor 647, 1/1000, Thermo Fisher Scientific, A-21235) diluted in 2% (w/v) bovine serum albumin (Sigma-Aldrich, St. Louis, MO, USA) in phosphate-buffered saline were incubated with samples for 1 h in the dark at room temperature. Samples were washed three times with IF buffer, and then nuclei were labeled with DAPI (4′,6-diamidino-2-phenylindole; 1 µg/ml, 5 min, Molecular Probes, Eugene, OR, USA). Samples were washed three times with phosphate-buffered saline, then mounted on microscope slides using mounting media (Dako, Mississauga, ON, Canada), and air-dried for at least 30 min. Samples were imaged using a fluorescence microscope (Leica DMI4000B).

### Western blotting

Western blot analysis followed published procedures^70^. In brief, crude extracts were separated by sodium dodecyl sulfate–polyacrylamide gel electrophoresis (SDS-PAGE) and blotted onto nitrocellulose membranes. Blocked filters were probed with antibodies against acetyl-α-tubulin K40 (1/10,000, Sigma-Aldrich, St. Louis, MO, USA, #T7451), α-tubulin (1/1000, Santa Cruz, sc-5286), phospho-AKT Ser473 (1/2000, Santa Cruz, sc-7985), pan-AKT (1/1500, Cell Signaling, #9272), HDAC6 (1/1000, Santa Cruz, sc-11420), EGFR (1/1000, Santa Cruz, sc-03), phospho-ERK1/2 Thr202/Tyr204 (1/2000, Cell Signaling, #9106), pan-ERK1/2 (1/2000, Cell Signaling, #4695), and actin (1/100,000, Chemicon, MAB1501). Signals for enhanced chemiluminescence were acquired with a Bio-Rad ChemiDoc™ MP imaging system and quantified.

### MTT assay

U251N cells were seeded in 24-well plates (Sarstedt, Nümbrecht, Germany) at 50,000 cells per well in 300 µl media and cultured for 24 h. Cells were treated with sahaquine (0.001, 1, 5, 10, 25, and 50 µM), TMZ (50, 100, 200, 300, 400, and 500 µM), quercetin (10, 50, 100, and 200 µM), or SAHA (0.1, 1, 2, 5, 8, 10, and 50 µM) for 72 h. Combination treatments included increasing concentrations of sahaquine (1, 10, and 50 µM) with a fixed concentration of quercetin (100 µM), or increasing concentrations of quercetin (10, 100, and 200 µM) with a fixed concentration of sahaquine (10 µM) for 72 h. Following treatment, MTT (Sigma-Aldrich, St. Louis, MO, USA) dissolved in phosphate-buffered saline was added to cells (0.5 mg/ml) for 1 h at 37 °C. After MTT-containing media were removed, dimethyl sulfoxide (0.5 ml) was added to each well to lyse cells and dissolve formazan. Wells were sampled in triplicate and the optical density was measured at 595 nm using a microplate reader (Asys UVM 340, Biochrom, Holliston, MA, USA).

### Gelatin zymography

U251N cells were seeded in 60-mm tissue culture dishes (Thermo Fisher Scientific, Rochester, NY, USA) at 1,500,000 cells per dish in 3 ml media and cultured for 24 h. Cells were treated in serum-deprived DMEM for 24 h. Following treatment, culture media were collected and concentrated 15-fold using 30 kDa centrifugal filters (Millipore, Cork, Ireland) following the manufacturer’s recommendations. Concentrated media were separated by SDS-PAGE using gelatin (0.1%, w/v) and acrylamide (7.5%, w/v) gels under non-reducing conditions. Gels were washed for 30 min in renaturing solution (2.5% (v/v) Triton X-100 in double-distilled water) and 30 min in developing buffer (50 mM Tris, pH 7.8; 1% (v/v) Triton X-100; 1 μM ZnCl_2_, 5 mM CaCl_2_, adjusted to pH 7.45). Gels were then incubated in fresh developing buffer at 37 °C overnight. Gels were stained with 0.5% (w/v) Coomassie Blue G250 (Bio-Rad, Richmond, CA, USA) dissolved in 40% (v/v) ethanol and 10% (v/v) acetic acid for 1 h, and then destained in 40% ethanol and 10% acetic acid diluted in double-distilled water, until clear bands appeared. Quantification of MMP-9 and MMP-2 abundance (as band area) was done in ImageJ.

### Tumoroid viability

U251N tumoroids were prepared using the liquid overlay system^79^. The 96-well cell culture plates were coated with 75 µl of 2% (w/v) agarose (Life Technologies, Gaithersburg, MD, USA) dissolved in serum-deprived DMEM. The agarose was cooled for 30 min, then cells were seeded at 5,000 cells per well in 200 µl media, and cultured for 4 days before treatment. Cells were treated for 7 days, and then imaged using a microscope (Leica DMI4000B). The surface area of tumoroids was analyzed in ImageJ.

### Calcein-AM uptake

U251N cells were seeded in 96-well black plates at 5,000 cells per well in 0.1 ml media and cultured for 24 h before treatment. Cyclosporine A (Calbiochem, Toronto, Canada) served as a positive control for the inhibition of P-glycoprotein. Following treatment, cells were incubated in phenol-free Hanks’ balanced Salt solution containing calcein-AM (0.5 µM, Thermo Fisher Scientific, Eugene, OR, USA) and propidium iodide (3 µM, Sigma-Aldrich, St. Louis, MO, USA) for 30 min at 37 °C. The media were replaced with fresh Hanks’ balanced salt solution and cells were imaged using a fluorescence microscope (Leica DMI4000B). Cells positively labeled with propidium iodide were excluded from the analysis.

### Statistics

Experiments were performed independently at least three times. Unless otherwise indicated, data are shown as mean (SD). Normality of data distribution was assessed by the Shapiro–Wilk test. For sample sizes larger than 30, the Central Limit Theorem allows the assumption of normal distribution. Equality of variances was assessed by Levene’s test. If the assumptions of normality and homogeneity of variance were met, two-tailed one-way analysis of variance (ANOVA) with Tukey–Kramer’s post hoc test were performed. If homogeneity of variance was not observed, Welch’s ANOVA with the Games–Howell post hoc test were used. A *p* value smaller than 0.05 was considered statistically significant: *p < 0.05, **p < 0.01, and ***p < 0.001.

## Electronic supplementary material

Supplemental Figures and Table
