# Supplementary material for: Inhibition of glioblastoma cell proliferation, invasion, and mechanism of action of a novel hydroxamic acid hybrid molecule
Source: Cell Death Discov. 2018 Sep 26;4:41. doi: 10.1038/s41420-018-0103-0 (PMC6158288; doi:10.1038/s41420-018-0103-0)
Supplement: Supplementary file 1 — Supplemental Figures and Table [file 41420_2018_103_MOESM1_ESM.pptx]

## Slide 1
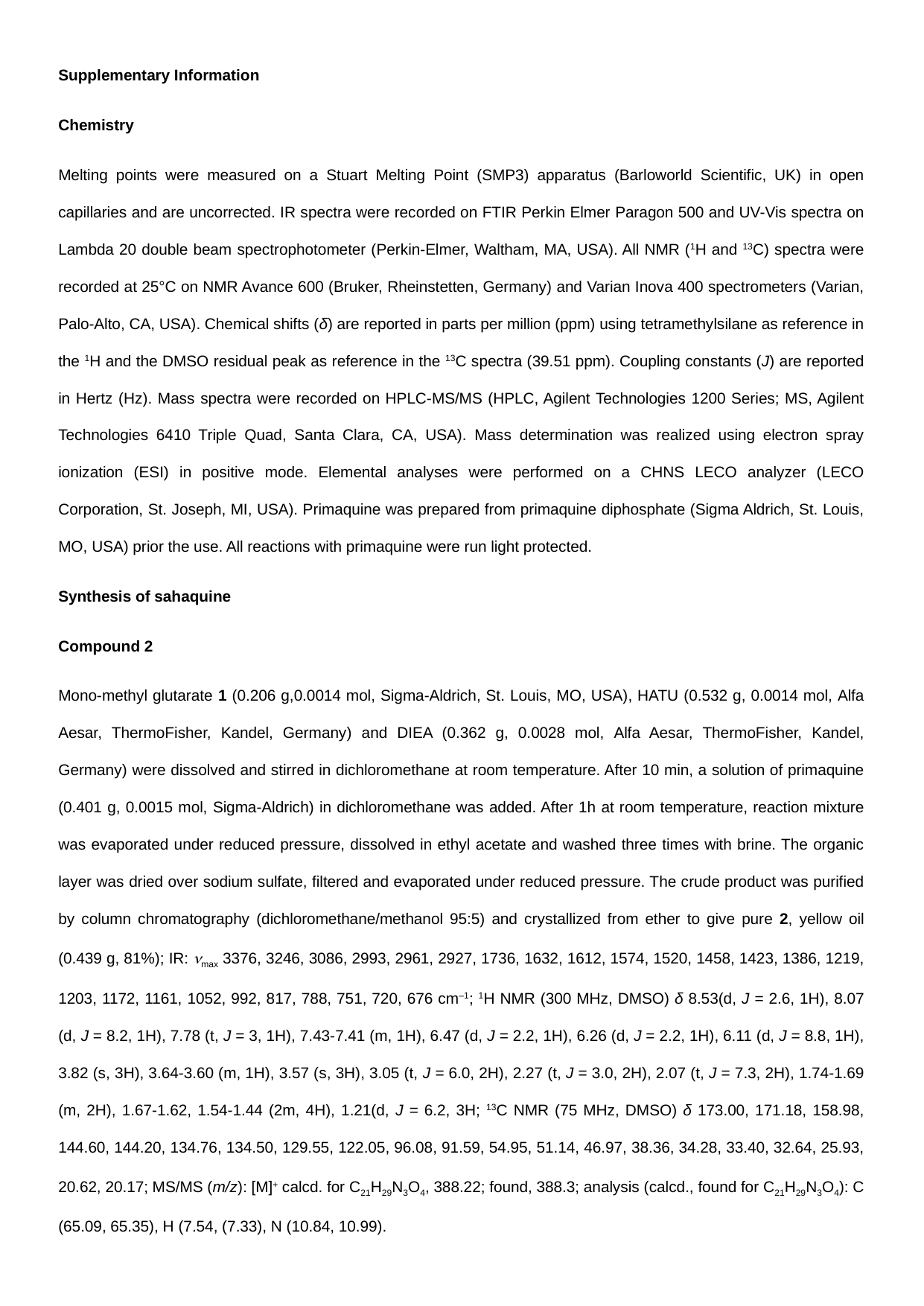

Supplementary Information
Chemistry
Melting points were measured on a Stuart Melting Point (SMP3) apparatus (Barloworld Scientific, UK) in open capillaries and are uncorrected. IR spectra were recorded on FTIR Perkin Elmer Paragon 500 and UV-Vis spectra on Lambda 20 double beam spectrophotometer (Perkin-Elmer, Waltham, MA, USA). All NMR (1H and 13C) spectra were recorded at 25°C on NMR Avance 600 (Bruker, Rheinstetten, Germany) and Varian Inova 400 spectrometers (Varian, Palo-Alto, CA, USA). Chemical shifts (δ) are reported in parts per million (ppm) using tetramethylsilane as reference in the 1H and the DMSO residual peak as reference in the 13C spectra (39.51 ppm). Coupling constants (J) are reported in Hertz (Hz). Mass spectra were recorded on HPLC-MS/MS (HPLC, Agilent Technologies 1200 Series; MS, Agilent Technologies 6410 Triple Quad, Santa Clara, CA, USA). Mass determination was realized using electron spray ionization (ESI) in positive mode. Elemental analyses were performed on a CHNS LECO analyzer (LECO Corporation, St. Joseph, MI, USA). Primaquine was prepared from primaquine diphosphate (Sigma Aldrich, St. Louis, MO, USA) prior the use. All reactions with primaquine were run light protected.
Synthesis of sahaquine
Compound 2
Mono-methyl glutarate 1 (0.206 g,0.0014 mol, Sigma-Aldrich, St. Louis, MO, USA), HATU (0.532 g, 0.0014 mol, Alfa Aesar, ThermoFisher, Kandel, Germany) and DIEA (0.362 g, 0.0028 mol, Alfa Aesar, ThermoFisher, Kandel, Germany) were dissolved and stirred in dichloromethane at room temperature. After 10 min, a solution of primaquine (0.401 g, 0.0015 mol, Sigma-Aldrich) in dichloromethane was added. After 1h at room temperature, reaction mixture was evaporated under reduced pressure, dissolved in ethyl acetate and washed three times with brine. The organic layer was dried over sodium sulfate, filtered and evaporated under reduced pressure. The crude product was purified by column chromatography (dichloromethane/methanol 95:5) and crystallized from ether to give pure 2, yellow oil (0.439 g, 81%); IR: max 3376, 3246, 3086, 2993, 2961, 2927, 1736, 1632, 1612, 1574, 1520, 1458, 1423, 1386, 1219, 1203, 1172, 1161, 1052, 992, 817, 788, 751, 720, 676 cm–1; 1H NMR (300 MHz, DMSO) δ 8.53(d, J = 2.6, 1H), 8.07 (d, J = 8.2, 1H), 7.78 (t, J = 3, 1H), 7.43-7.41 (m, 1H), 6.47 (d, J = 2.2, 1H), 6.26 (d, J = 2.2, 1H), 6.11 (d, J = 8.8, 1H), 3.82 (s, 3H), 3.64-3.60 (m, 1H), 3.57 (s, 3H), 3.05 (t, J = 6.0, 2H), 2.27 (t, J = 3.0, 2H), 2.07 (t, J = 7.3, 2H), 1.74-1.69 (m, 2H), 1.67-1.62, 1.54-1.44 (2m, 4H), 1.21(d, J = 6.2, 3H; 13C NMR (75 MHz, DMSO) δ 173.00, 171.18, 158.98, 144.60, 144.20, 134.76, 134.50, 129.55, 122.05, 96.08, 91.59, 54.95, 51.14, 46.97, 38.36, 34.28, 33.40, 32.64, 25.93, 20.62, 20.17; MS/MS (m/z): [M]+ calcd. for C21H29N3O4, 388.22; found, 388.3; analysis (calcd., found for C21H29N3O4): C (65.09, 65.35), H (7.54, (7.33), N (10.84, 10.99).

## Slide 2
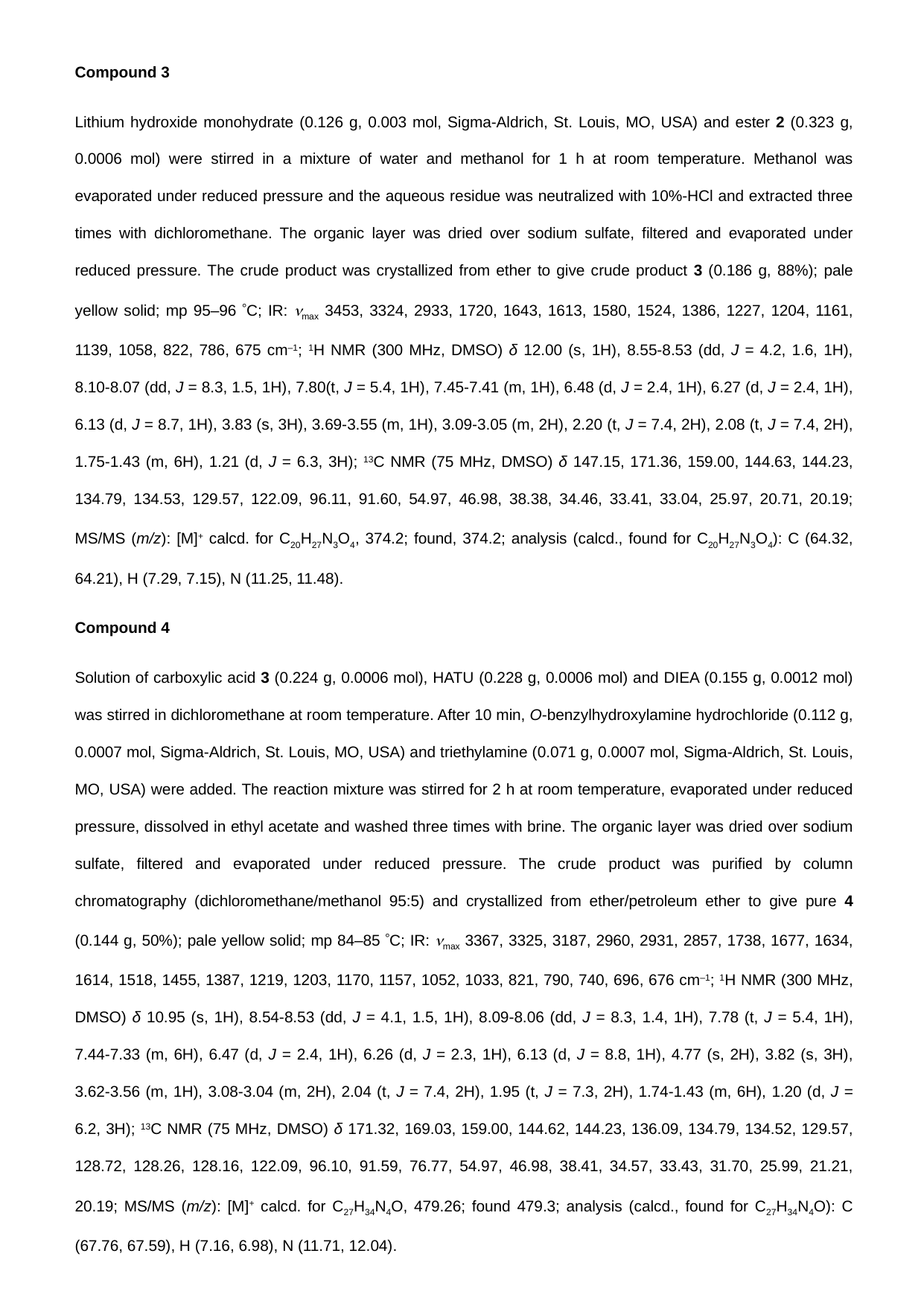

Compound 3
Lithium hydroxide monohydrate (0.126 g, 0.003 mol, Sigma-Aldrich, St. Louis, MO, USA) and ester 2 (0.323 g, 0.0006 mol) were stirred in a mixture of water and methanol for 1 h at room temperature. Methanol was evaporated under reduced pressure and the aqueous residue was neutralized with 10%-HCl and extracted three times with dichloromethane. The organic layer was dried over sodium sulfate, filtered and evaporated under reduced pressure. The crude product was crystallized from ether to give crude product 3 (0.186 g, 88%); pale yellow solid; mp 95–96 °C; IR: max 3453, 3324, 2933, 1720, 1643, 1613, 1580, 1524, 1386, 1227, 1204, 1161, 1139, 1058, 822, 786, 675 cm–1; 1H NMR (300 MHz, DMSO) δ 12.00 (s, 1H), 8.55-8.53 (dd, J = 4.2, 1.6, 1H), 8.10-8.07 (dd, J = 8.3, 1.5, 1H), 7.80(t, J = 5.4, 1H), 7.45-7.41 (m, 1H), 6.48 (d, J = 2.4, 1H), 6.27 (d, J = 2.4, 1H), 6.13 (d, J = 8.7, 1H), 3.83 (s, 3H), 3.69-3.55 (m, 1H), 3.09-3.05 (m, 2H), 2.20 (t, J = 7.4, 2H), 2.08 (t, J = 7.4, 2H), 1.75-1.43 (m, 6H), 1.21 (d, J = 6.3, 3H); 13C NMR (75 MHz, DMSO) δ 147.15, 171.36, 159.00, 144.63, 144.23, 134.79, 134.53, 129.57, 122.09, 96.11, 91.60, 54.97, 46.98, 38.38, 34.46, 33.41, 33.04, 25.97, 20.71, 20.19; MS/MS (m/z): [M]+ calcd. for C20H27N3O4, 374.2; found, 374.2; analysis (calcd., found for C20H27N3O4): C (64.32, 64.21), H (7.29, 7.15), N (11.25, 11.48).
Compound 4
Solution of carboxylic acid 3 (0.224 g, 0.0006 mol), HATU (0.228 g, 0.0006 mol) and DIEA (0.155 g, 0.0012 mol) was stirred in dichloromethane at room temperature. After 10 min, O-benzylhydroxylamine hydrochloride (0.112 g, 0.0007 mol, Sigma-Aldrich, St. Louis, MO, USA) and triethylamine (0.071 g, 0.0007 mol, Sigma-Aldrich, St. Louis, MO, USA) were added. The reaction mixture was stirred for 2 h at room temperature, evaporated under reduced pressure, dissolved in ethyl acetate and washed three times with brine. The organic layer was dried over sodium sulfate, filtered and evaporated under reduced pressure. The crude product was purified by column chromatography (dichloromethane/methanol 95:5) and crystallized from ether/petroleum ether to give pure 4 (0.144 g, 50%); pale yellow solid; mp 84–85 °C; IR: max 3367, 3325, 3187, 2960, 2931, 2857, 1738, 1677, 1634, 1614, 1518, 1455, 1387, 1219, 1203, 1170, 1157, 1052, 1033, 821, 790, 740, 696, 676 cm–1; 1H NMR (300 MHz, DMSO) δ 10.95 (s, 1H), 8.54-8.53 (dd, J = 4.1, 1.5, 1H), 8.09-8.06 (dd, J = 8.3, 1.4, 1H), 7.78 (t, J = 5.4, 1H), 7.44-7.33 (m, 6H), 6.47 (d, J = 2.4, 1H), 6.26 (d, J = 2.3, 1H), 6.13 (d, J = 8.8, 1H), 4.77 (s, 2H), 3.82 (s, 3H), 3.62-3.56 (m, 1H), 3.08-3.04 (m, 2H), 2.04 (t, J = 7.4, 2H), 1.95 (t, J = 7.3, 2H), 1.74-1.43 (m, 6H), 1.20 (d, J = 6.2, 3H); 13C NMR (75 MHz, DMSO) δ 171.32, 169.03, 159.00, 144.62, 144.23, 136.09, 134.79, 134.52, 129.57, 128.72, 128.26, 128.16, 122.09, 96.10, 91.59, 76.77, 54.97, 46.98, 38.41, 34.57, 33.43, 31.70, 25.99, 21.21, 20.19; MS/MS (m/z): [M]+ calcd. for C27H34N4O, 479.26; found 479.3; analysis (calcd., found for C27H34N4O): C (67.76, 67.59), H (7.16, 6.98), N (11.71, 12.04).

## Slide 3
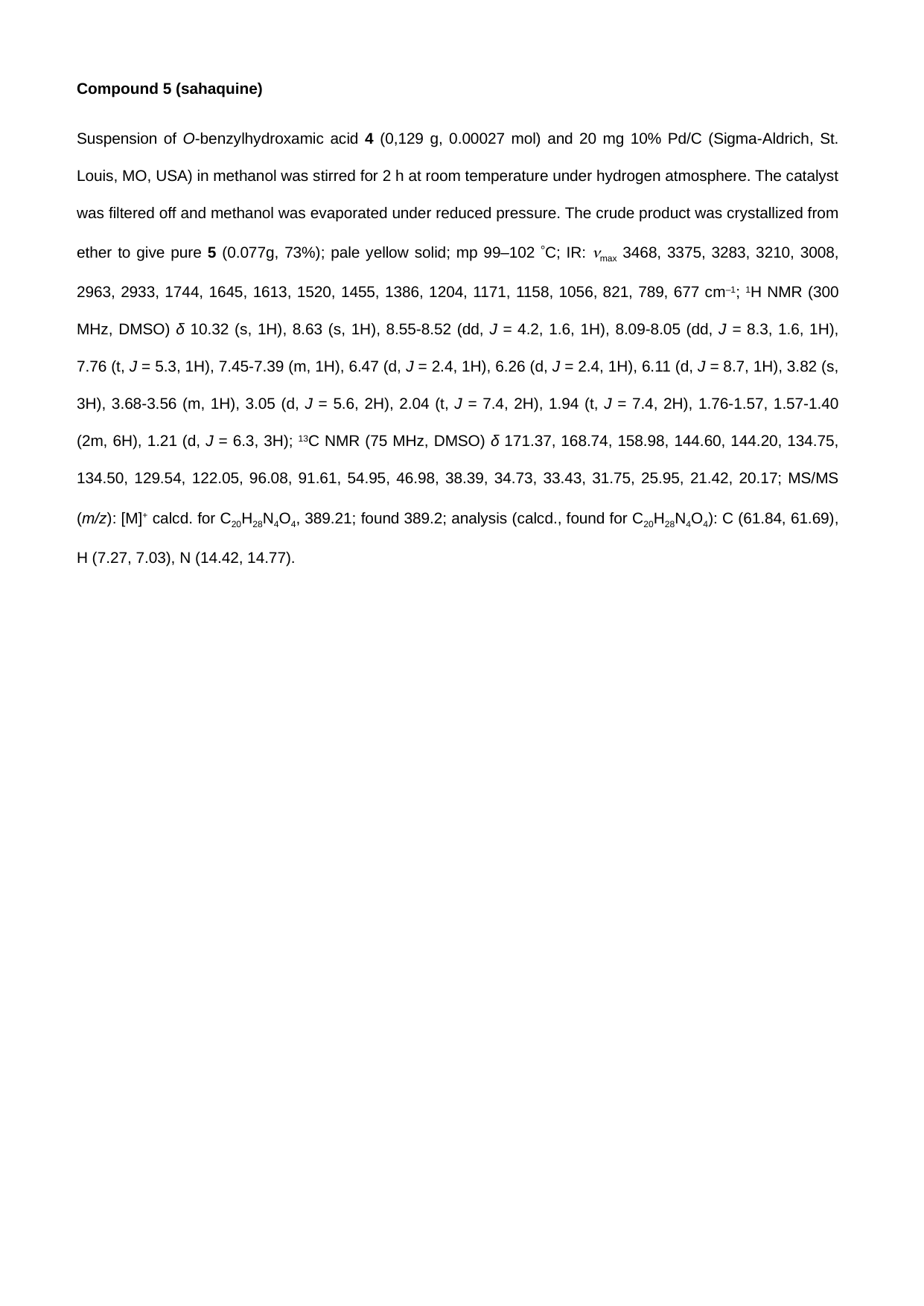

Compound 5 (sahaquine)
Suspension of O-benzylhydroxamic acid 4 (0,129 g, 0.00027 mol) and 20 mg 10% Pd/C (Sigma-Aldrich, St. Louis, MO, USA) in methanol was stirred for 2 h at room temperature under hydrogen atmosphere. The catalyst was filtered off and methanol was evaporated under reduced pressure. The crude product was crystallized from ether to give pure 5 (0.077g, 73%); pale yellow solid; mp 99–102 °C; IR: max 3468, 3375, 3283, 3210, 3008, 2963, 2933, 1744, 1645, 1613, 1520, 1455, 1386, 1204, 1171, 1158, 1056, 821, 789, 677 cm–1; 1H NMR (300 MHz, DMSO) δ 10.32 (s, 1H), 8.63 (s, 1H), 8.55-8.52 (dd, J = 4.2, 1.6, 1H), 8.09-8.05 (dd, J = 8.3, 1.6, 1H), 7.76 (t, J = 5.3, 1H), 7.45-7.39 (m, 1H), 6.47 (d, J = 2.4, 1H), 6.26 (d, J = 2.4, 1H), 6.11 (d, J = 8.7, 1H), 3.82 (s, 3H), 3.68-3.56 (m, 1H), 3.05 (d, J = 5.6, 2H), 2.04 (t, J = 7.4, 2H), 1.94 (t, J = 7.4, 2H), 1.76-1.57, 1.57-1.40 (2m, 6H), 1.21 (d, J = 6.3, 3H); 13C NMR (75 MHz, DMSO) δ 171.37, 168.74, 158.98, 144.60, 144.20, 134.75, 134.50, 129.54, 122.05, 96.08, 91.61, 54.95, 46.98, 38.39, 34.73, 33.43, 31.75, 25.95, 21.42, 20.17; MS/MS (m/z): [M]+ calcd. for C20H28N4O4, 389.21; found 389.2; analysis (calcd., found for C20H28N4O4): C (61.84, 61.69), H (7.27, 7.03), N (14.42, 14.77).

## Slide 4
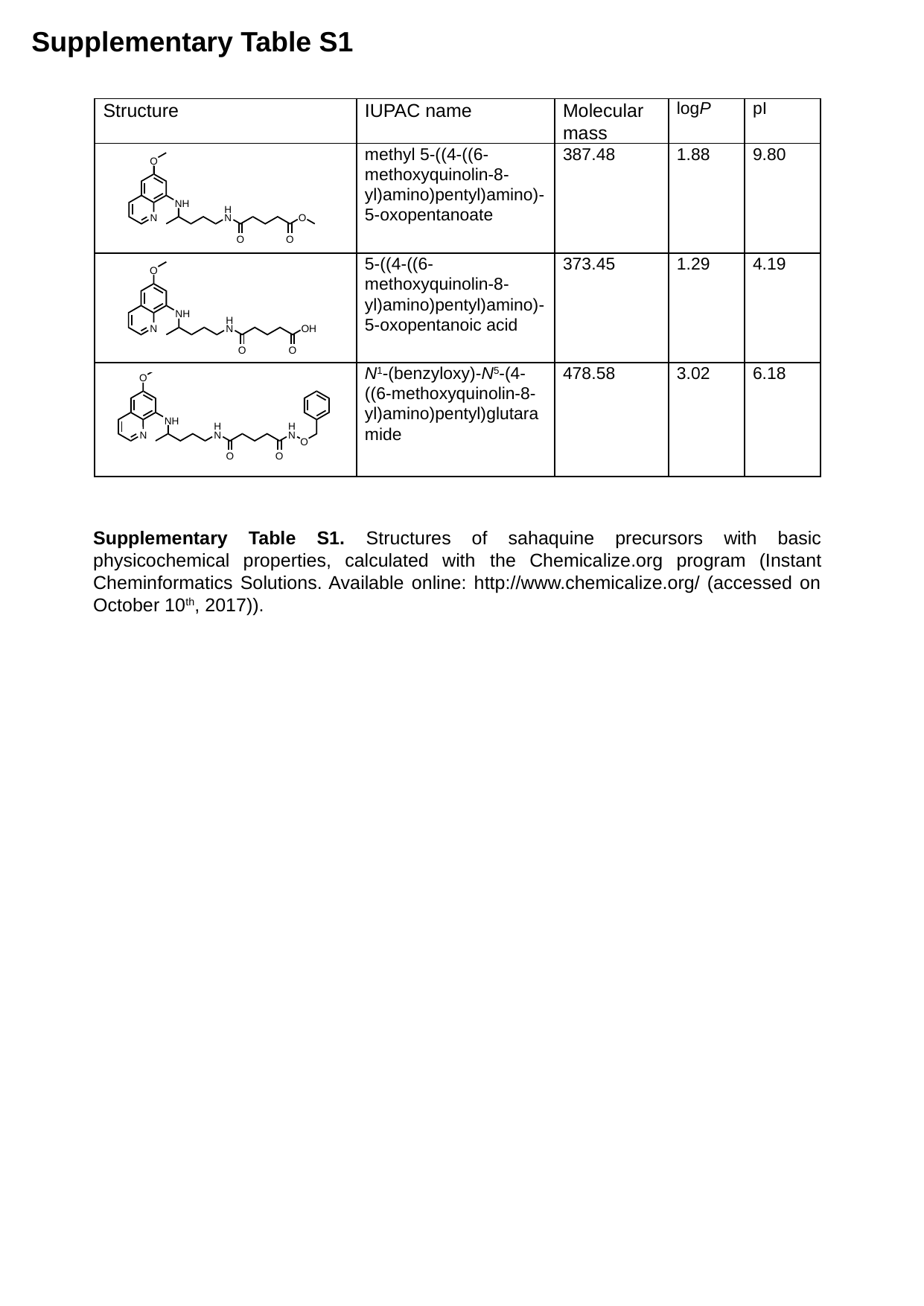

Supplementary Table S1
| Structure | IUPAC name | Molecular mass | logP | pI |
| --- | --- | --- | --- | --- |
| | methyl 5-((4-((6-methoxyquinolin-8-yl)amino)pentyl)amino)-5-oxopentanoate | 387.48 | 1.88 | 9.80 |
| | 5-((4-((6-methoxyquinolin-8-yl)amino)pentyl)amino)-5-oxopentanoic acid | 373.45 | 1.29 | 4.19 |
| | N1-(benzyloxy)-N5-(4-((6-methoxyquinolin-8-yl)amino)pentyl)glutaramide | 478.58 | 3.02 | 6.18 |
Supplementary Table S1. Structures of sahaquine precursors with basic physicochemical properties, calculated with the Chemicalize.org program (Instant Cheminformatics Solutions. Available online: http://www.chemicalize.org/ (accessed on October 10th, 2017)).

## Slide 5
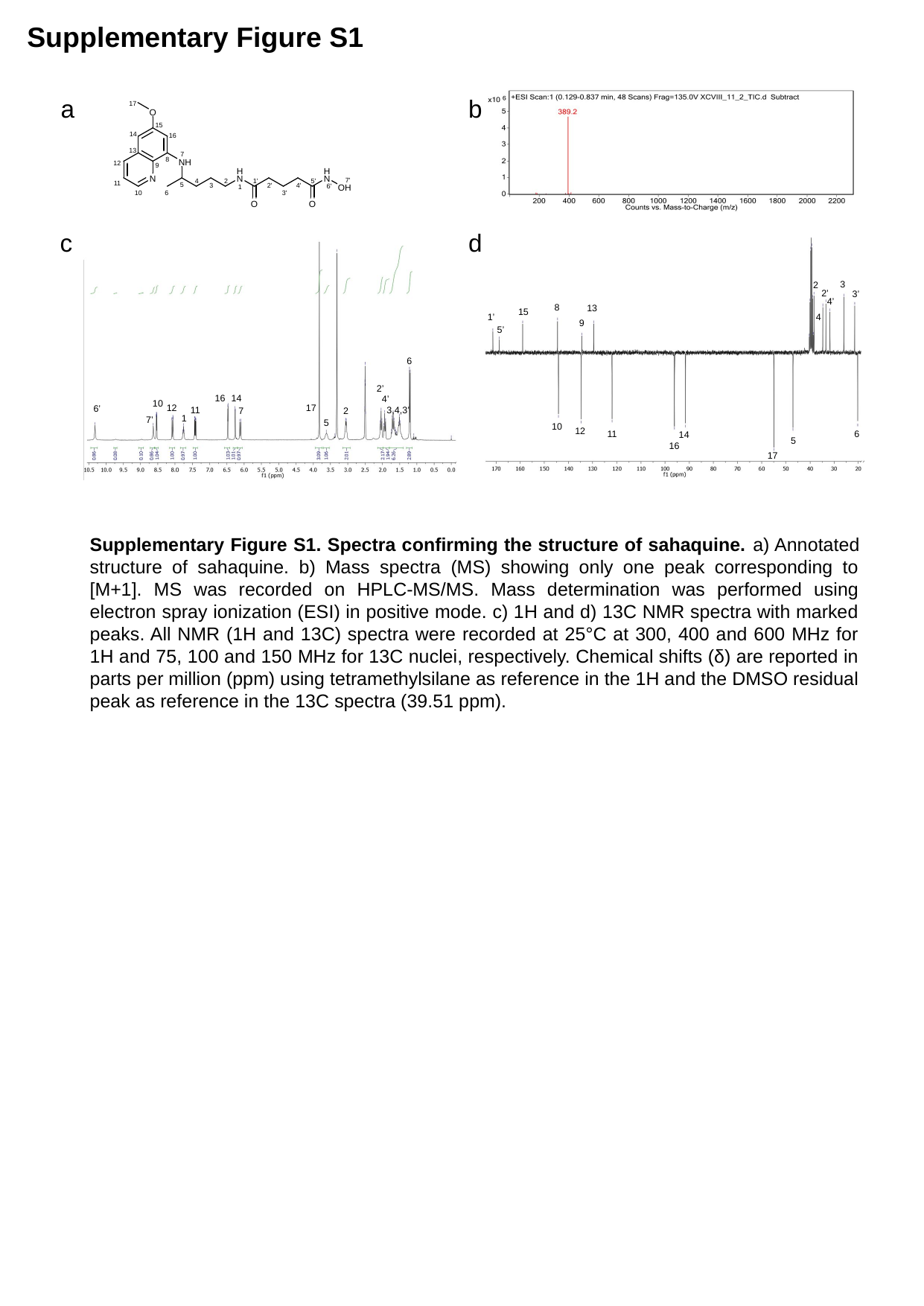

Supplementary Figure S1
a
b
c
d
3
2
2’
3’
4’
8
13
15
1’
4
9
5’
6
2’
16
14
4’
10
12
17
6’
3,4,3’
11
2
7
1
7’
5
10
12
11
6
14
5
16
17
Supplementary Figure S1. Spectra confirming the structure of sahaquine. a) Annotated structure of sahaquine. b) Mass spectra (MS) showing only one peak corresponding to [M+1]. MS was recorded on HPLC-MS/MS. Mass determination was performed using electron spray ionization (ESI) in positive mode. c) 1H and d) 13C NMR spectra with marked peaks. All NMR (1H and 13C) spectra were recorded at 25°C at 300, 400 and 600 MHz for 1H and 75, 100 and 150 MHz for 13C nuclei, respectively. Chemical shifts (δ) are reported in parts per million (ppm) using tetramethylsilane as reference in the 1H and the DMSO residual peak as reference in the 13C spectra (39.51 ppm).

## Slide 6
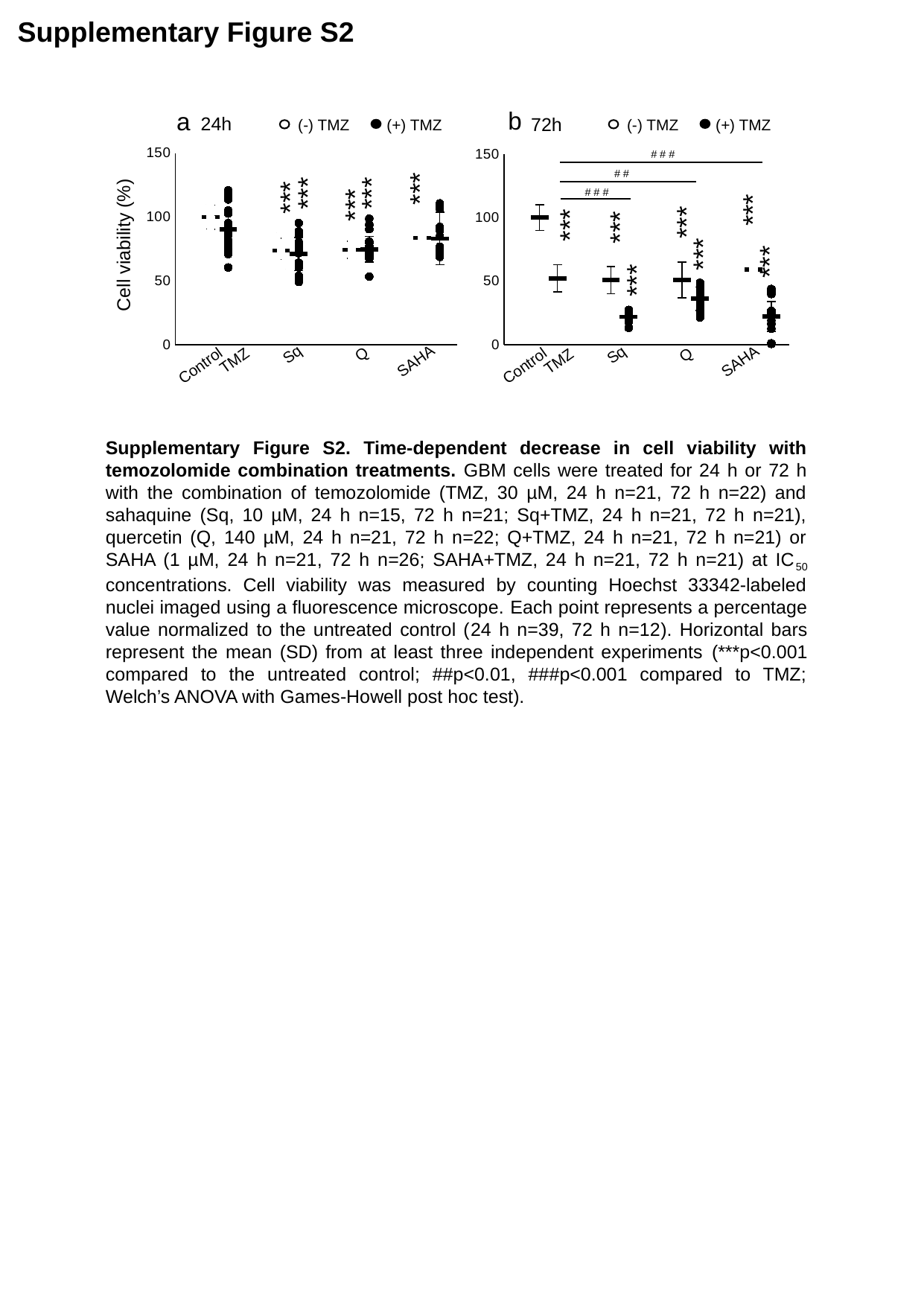

Supplementary Figure S2
b
a
24h
72h
(-) TMZ
(+) TMZ
(-) TMZ
(+) TMZ
### Chart
| Category | Control | TMZ | Sq | Q | SAHA | TMZ+Sq | TMZ+Q | TMZ+SAHA | Control | TMZ | Sq | Q | SAHA | TMZ+Sq | TMZ+Q | TMZ+SAHA |
|---|---|---|---|---|---|---|---|---|---|---|---|---|---|---|---|---|
### Chart
| Category | Control | TMZ | Sq | Q | SAHA | TMZ+Sq | TMZ+Q | TMZ+SAHA | Control | TMZ | Sq | Q | SAHA | TMZ+Sq | TMZ+Q | TMZ+SAHA |
|---|---|---|---|---|---|---|---|---|---|---|---|---|---|---|---|---|# # #
***
# #
***
***
***
***
# # #
***
***
***
***
Cell viability (%)
***
***
***
Q
Q
Sq
Sq
SAHA
Control
TMZ
SAHA
Control
TMZ
Supplementary Figure S2. Time-dependent decrease in cell viability with temozolomide combination treatments. GBM cells were treated for 24 h or 72 h with the combination of temozolomide (TMZ, 30 µM, 24 h n=21, 72 h n=22) and sahaquine (Sq, 10 µM, 24 h n=15, 72 h n=21; Sq+TMZ, 24 h n=21, 72 h n=21), quercetin (Q, 140 µM, 24 h n=21, 72 h n=22; Q+TMZ, 24 h n=21, 72 h n=21) or SAHA (1 µM, 24 h n=21, 72 h n=26; SAHA+TMZ, 24 h n=21, 72 h n=21) at IC50 concentrations. Cell viability was measured by counting Hoechst 33342-labeled nuclei imaged using a fluorescence microscope. Each point represents a percentage value normalized to the untreated control (24 h n=39, 72 h n=12). Horizontal bars represent the mean (SD) from at least three independent experiments (***p<0.001 compared to the untreated control; ##p<0.01, ###p<0.001 compared to TMZ; Welch’s ANOVA with Games-Howell post hoc test).

## Slide 7
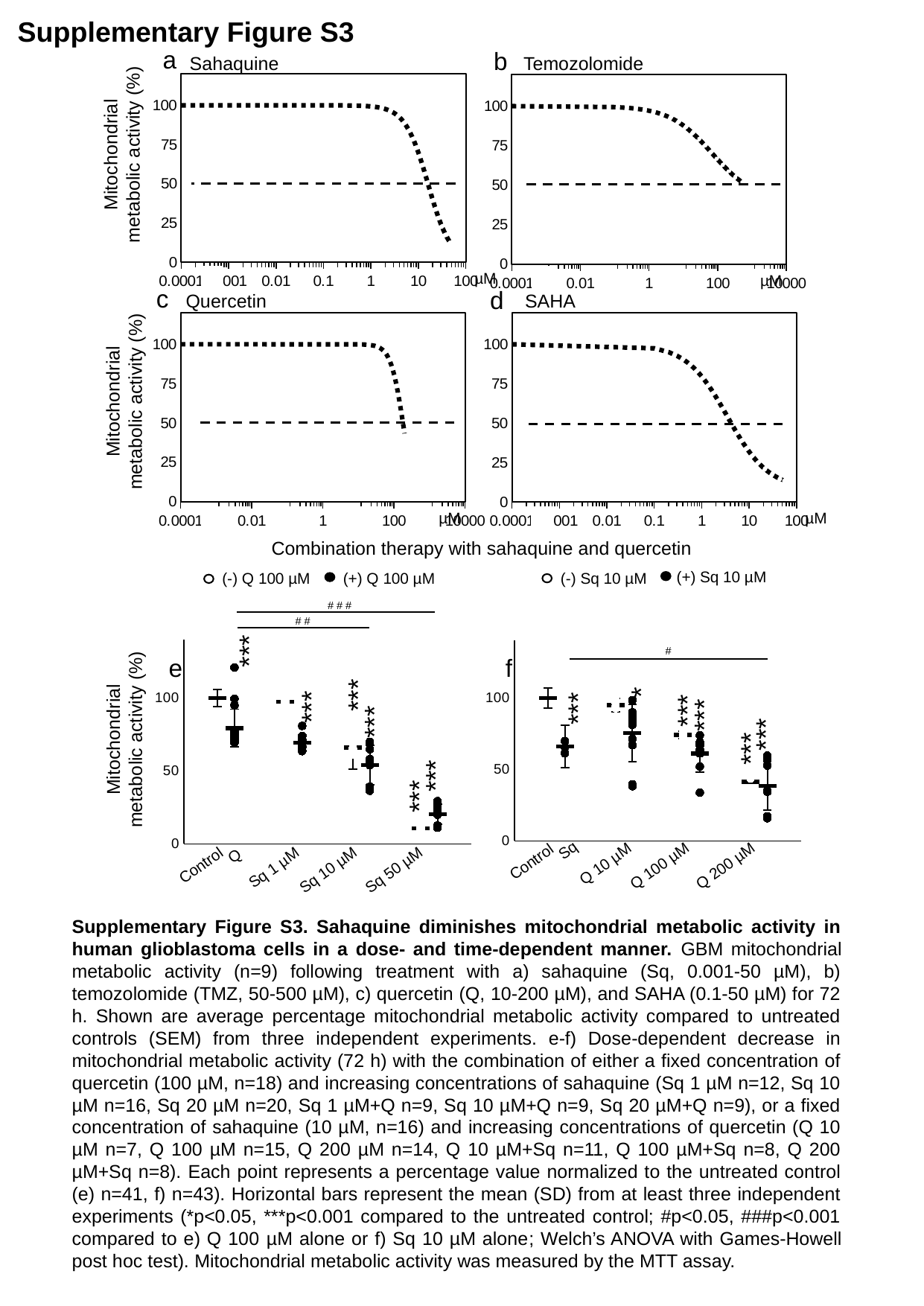

Supplementary Figure S3
a
b
Sahaquine
Temozolomide
### Chart
| Category | 72h: IC50 = 2 µM | | |
|---|---|---|---|
### Chart
| Category | 72h: IC50 = 23.0 µM | |
|---|---|---|Mitochondrial metabolic activity (%)
µM
µM
c
d
Quercetin
SAHA
### Chart
| Category | 72h: IC50 = 139 µM | |
|---|---|---|
### Chart
| Category | 72h: IC50 = 1 µM | |
|---|---|---|Mitochondrial metabolic activity (%)
µM
µM
Combination therapy with sahaquine and quercetin
(+) Sq 10 µM
(-) Q 100 µM
(+) Q 100 µM
(-) Sq 10 µM
# # #
# #
***
### Chart
| Category | Control | Q100 uM | Control | Q100 uM | Sq 1 uM | Sq 10 uM | Sq 50 uM | Sq 1 uM+Q100 | Sq10 uM + Q100 uM | Sq 20 uM | Sq 1 uM | Sq 10 uM | Sq 50 uM | Sq 1 uM+Q100 | Sq10 uM + Q100 uM | Sq 20 uM |
|---|---|---|---|---|---|---|---|---|---|---|---|---|---|---|---|---|
### Chart
| Category | Control | Sq10 uM | Control | Sq10 uM | Q 10 uM | Q 100 uM | Q 200 uM | Sq10+Q 10 uM | Sq10+Q 100 uM | Sq10+Q 200 uM | Q 10 uM | Q 100 uM | Q 200 uM | Sq10+Q 10 uM | Sq10+Q 100 uM | Sq10+Q 200 uM |
|---|---|---|---|---|---|---|---|---|---|---|---|---|---|---|---|---|#
e
f
*
***
***
***
***
***
***
***
Mitochondrial metabolic activity (%)
***
***
***
Sq
Q
Control
Control
Q 10 µM
Q 100 µM
Q 200 µM
Sq 1 µM
Sq 10 µM
Sq 50 µM
Supplementary Figure S3. Sahaquine diminishes mitochondrial metabolic activity in human glioblastoma cells in a dose- and time-dependent manner. GBM mitochondrial metabolic activity (n=9) following treatment with a) sahaquine (Sq, 0.001-50 µM), b) temozolomide (TMZ, 50-500 µM), c) quercetin (Q, 10-200 µM), and SAHA (0.1-50 µM) for 72 h. Shown are average percentage mitochondrial metabolic activity compared to untreated controls (SEM) from three independent experiments. e-f) Dose-dependent decrease in mitochondrial metabolic activity (72 h) with the combination of either a fixed concentration of quercetin (100 µM, n=18) and increasing concentrations of sahaquine (Sq 1 µM n=12, Sq 10 µM n=16, Sq 20 µM n=20, Sq 1 µM+Q n=9, Sq 10 µM+Q n=9, Sq 20 µM+Q n=9), or a fixed concentration of sahaquine (10 µM, n=16) and increasing concentrations of quercetin (Q 10 µM n=7, Q 100 µM n=15, Q 200 µM n=14, Q 10 µM+Sq n=11, Q 100 µM+Sq n=8, Q 200 µM+Sq n=8). Each point represents a percentage value normalized to the untreated control (e) n=41, f) n=43). Horizontal bars represent the mean (SD) from at least three independent experiments (*p<0.05, ***p<0.001 compared to the untreated control; #p<0.05, ###p<0.001 compared to e) Q 100 µM alone or f) Sq 10 µM alone; Welch’s ANOVA with Games-Howell post hoc test). Mitochondrial metabolic activity was measured by the MTT assay.

## Slide 8
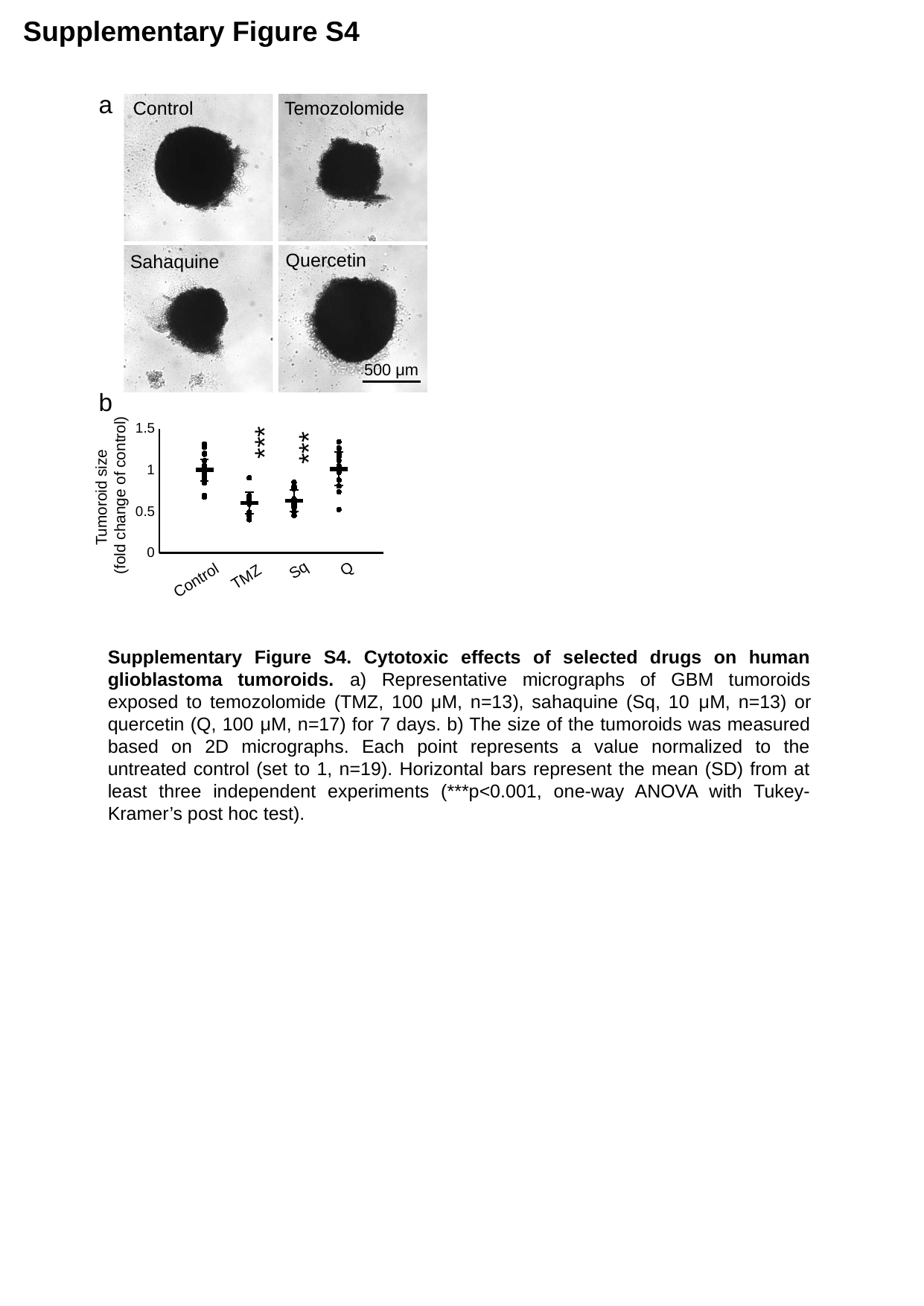

Supplementary Figure S4
a
Temozolomide
Control
Quercetin
Sahaquine
500 μm
b
***
### Chart
| Category | Control | TMZ 100 uM | Sahaq 10 μM | Quercetin 100 μM | Control | TMZ 100 uM | Sahaq 10 μM | Quercetin 100 μM |
|---|---|---|---|---|---|---|---|---|***
Tumoroid size
(fold change of control)
Q
Sq
Control
TMZ
Supplementary Figure S4. Cytotoxic effects of selected drugs on human glioblastoma tumoroids. a) Representative micrographs of GBM tumoroids exposed to temozolomide (TMZ, 100 μM, n=13), sahaquine (Sq, 10 μM, n=13) or quercetin (Q, 100 μM, n=17) for 7 days. b) The size of the tumoroids was measured based on 2D micrographs. Each point represents a value normalized to the untreated control (set to 1, n=19). Horizontal bars represent the mean (SD) from at least three independent experiments (***p<0.001, one-way ANOVA with Tukey-Kramer’s post hoc test).

## Slide 9
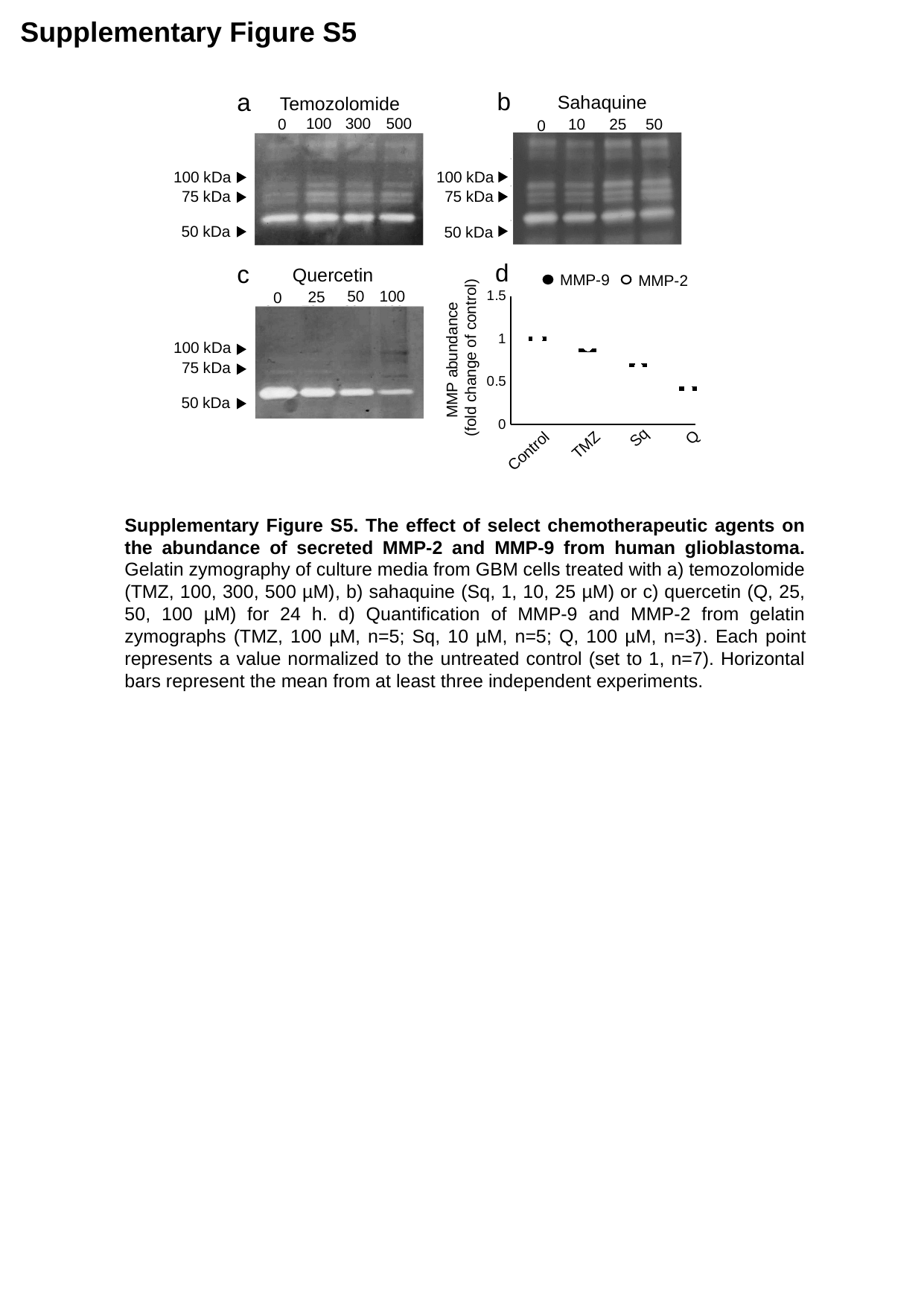

Supplementary Figure S5
b
a
Sahaquine
Temozolomide
500
300
100
50
25
10
0
0
100 kDa
100 kDa
75 kDa
75 kDa
50 kDa
50 kDa
d
c
Quercetin
MMP-9
MMP-2
100
50
25
0
### Chart
| Category | Control | TMZ 100 uM | Sahaq 10 μM | Quercetin 100 μM | Control | TMZ 100 uM | Sahaq 10 μM | Quercetin 100 μM | Control | TMZ 100 uM | Sahaq 10 μM | Quercetin 100 μM | Control | TMZ 100 uM | Sahaq 10 μM | Quercetin 100 μM |
|---|---|---|---|---|---|---|---|---|---|---|---|---|---|---|---|---|
100 kDa
MMP abundance
(fold change of control)
75 kDa
50 kDa
Q
Sq
TMZ
Control
Supplementary Figure S5. The effect of select chemotherapeutic agents on the abundance of secreted MMP-2 and MMP-9 from human glioblastoma. Gelatin zymography of culture media from GBM cells treated with a) temozolomide (TMZ, 100, 300, 500 µM), b) sahaquine (Sq, 1, 10, 25 µM) or c) quercetin (Q, 25, 50, 100 µM) for 24 h. d) Quantification of MMP-9 and MMP-2 from gelatin zymographs (TMZ, 100 µM, n=5; Sq, 10 µM, n=5; Q, 100 µM, n=3). Each point represents a value normalized to the untreated control (set to 1, n=7). Horizontal bars represent the mean from at least three independent experiments.

## Slide 10
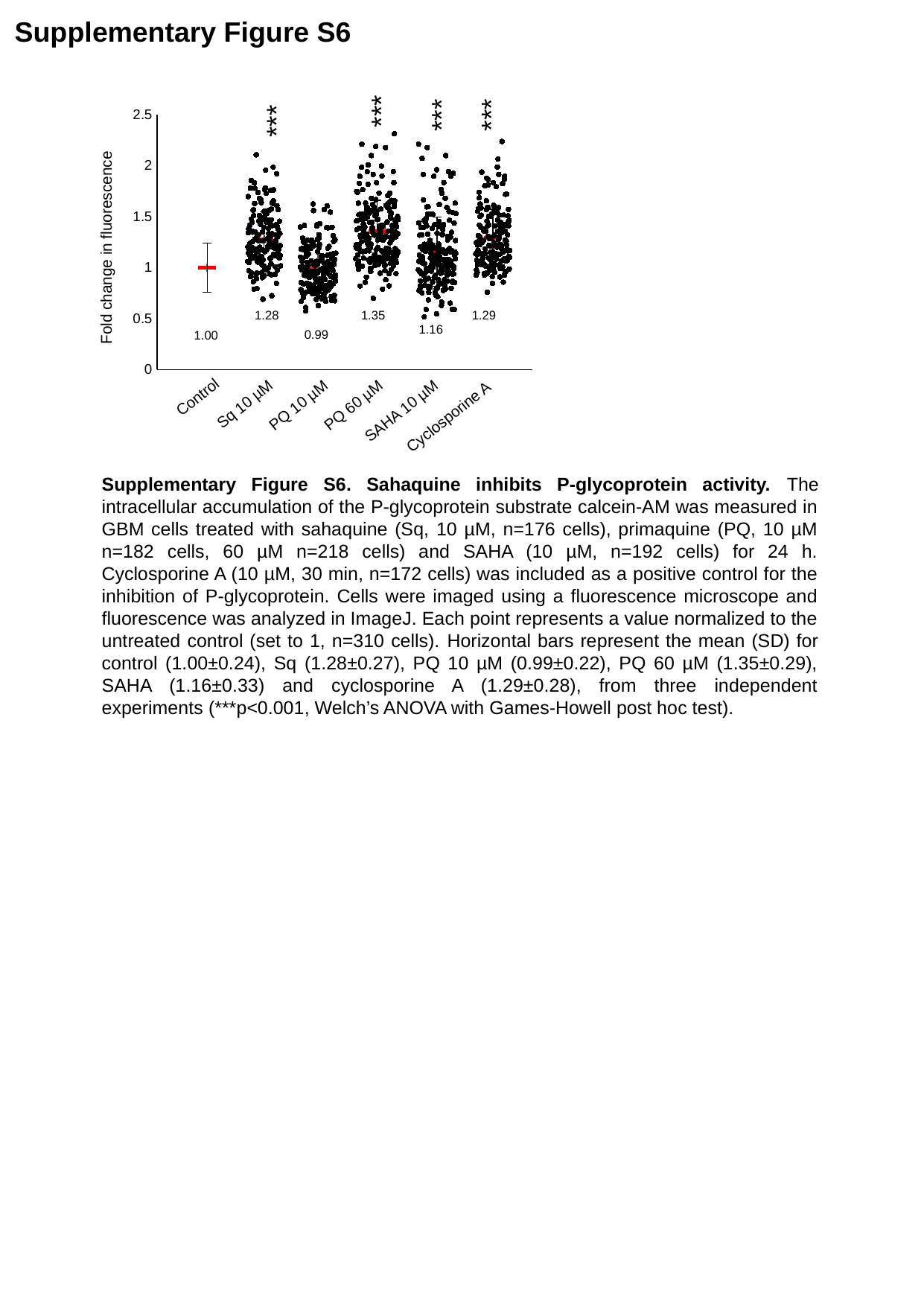

Supplementary Figure S6
***
***
***
***
### Chart
| Category | Control | Sq | SAHA | PQ 60 uM | Cyclosporine A | Control | Sq | SAHA | PQ 60 uM | Cyclosporine A | PQ 10 uM | PQ 10 uM |
|---|---|---|---|---|---|---|---|---|---|---|---|---|Fold change in fluorescence
1.28
1.29
1.35
1.16
0.99
1.00
Control
Sq 10 µM
PQ 60 µM
PQ 10 µM
SAHA 10 µM
Cyclosporine A
Supplementary Figure S6. Sahaquine inhibits P-glycoprotein activity. The intracellular accumulation of the P-glycoprotein substrate calcein-AM was measured in GBM cells treated with sahaquine (Sq, 10 µM, n=176 cells), primaquine (PQ, 10 µM n=182 cells, 60 µM n=218 cells) and SAHA (10 µM, n=192 cells) for 24 h. Cyclosporine A (10 µM, 30 min, n=172 cells) was included as a positive control for the inhibition of P-glycoprotein. Cells were imaged using a fluorescence microscope and fluorescence was analyzed in ImageJ. Each point represents a value normalized to the untreated control (set to 1, n=310 cells). Horizontal bars represent the mean (SD) for control (1.00±0.24), Sq (1.28±0.27), PQ 10 µM (0.99±0.22), PQ 60 µM (1.35±0.29), SAHA (1.16±0.33) and cyclosporine A (1.29±0.28), from three independent experiments (***p<0.001, Welch’s ANOVA with Games-Howell post hoc test).

## Slide 11
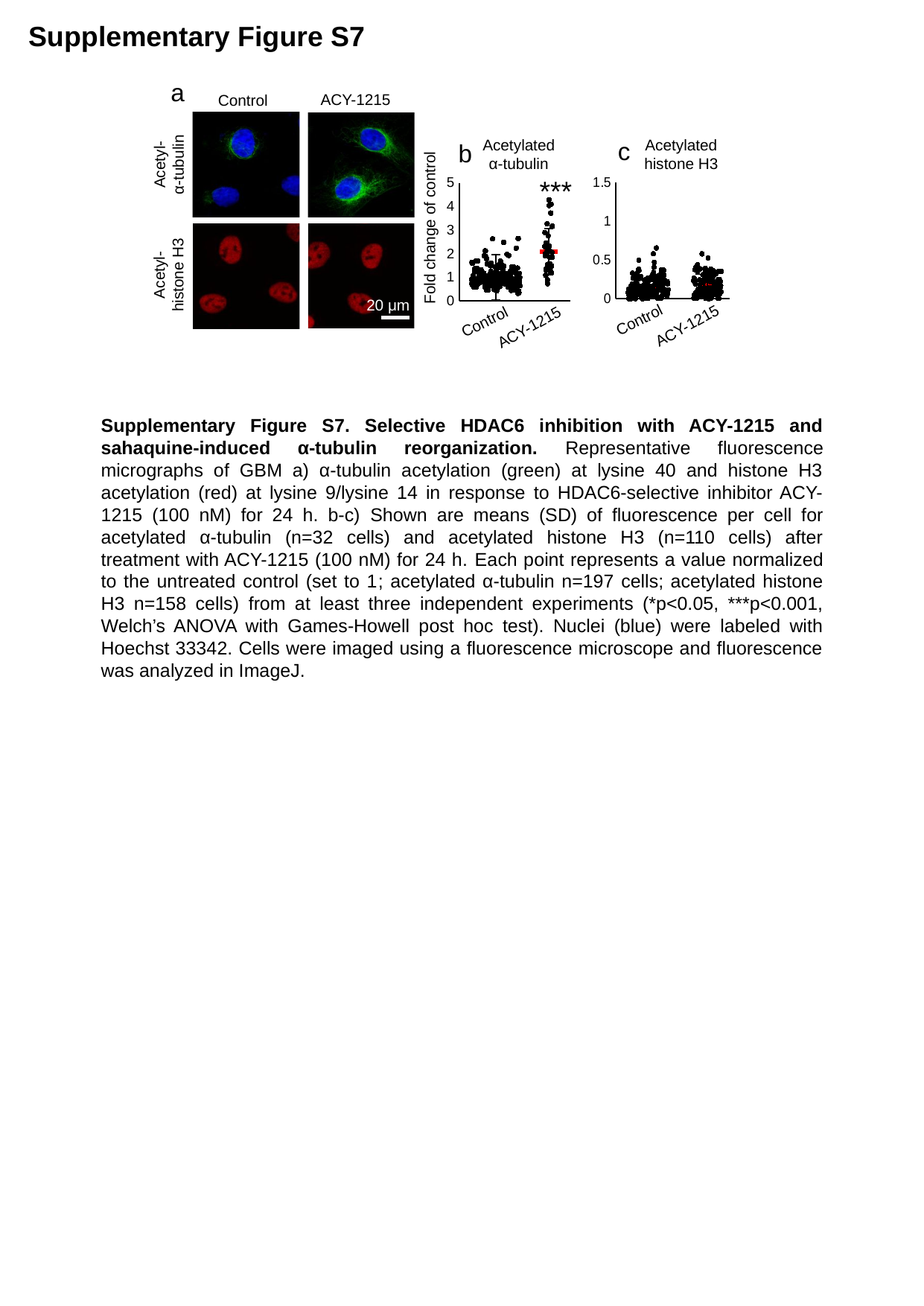

Supplementary Figure S7
a
ACY-1215
Control
c
Acetylated α-tubulin
Acetylated histone H3
b
Acetyl-
α-tubulin
***
### Chart
| Category | Control | ACY 100 nM | | | Control | ACY 100 nM | | | | | |
|---|---|---|---|---|---|---|---|---|---|---|---|
### Chart
| Category | Control | ACY 100 nM | | | Control | ACY 100 nM | | | | | |
|---|---|---|---|---|---|---|---|---|---|---|---|Fold change of control
Acetyl-
histone H3
20 μm
Control
Control
ACY-1215
ACY-1215
Supplementary Figure S7. Selective HDAC6 inhibition with ACY-1215 and sahaquine-induced α-tubulin reorganization. Representative fluorescence micrographs of GBM a) α-tubulin acetylation (green) at lysine 40 and histone H3 acetylation (red) at lysine 9/lysine 14 in response to HDAC6-selective inhibitor ACY-1215 (100 nM) for 24 h. b-c) Shown are means (SD) of fluorescence per cell for acetylated α-tubulin (n=32 cells) and acetylated histone H3 (n=110 cells) after treatment with ACY-1215 (100 nM) for 24 h. Each point represents a value normalized to the untreated control (set to 1; acetylated α-tubulin n=197 cells; acetylated histone H3 n=158 cells) from at least three independent experiments (*p<0.05, ***p<0.001, Welch’s ANOVA with Games-Howell post hoc test). Nuclei (blue) were labeled with Hoechst 33342. Cells were imaged using a fluorescence microscope and fluorescence was analyzed in ImageJ.

## Slide 12
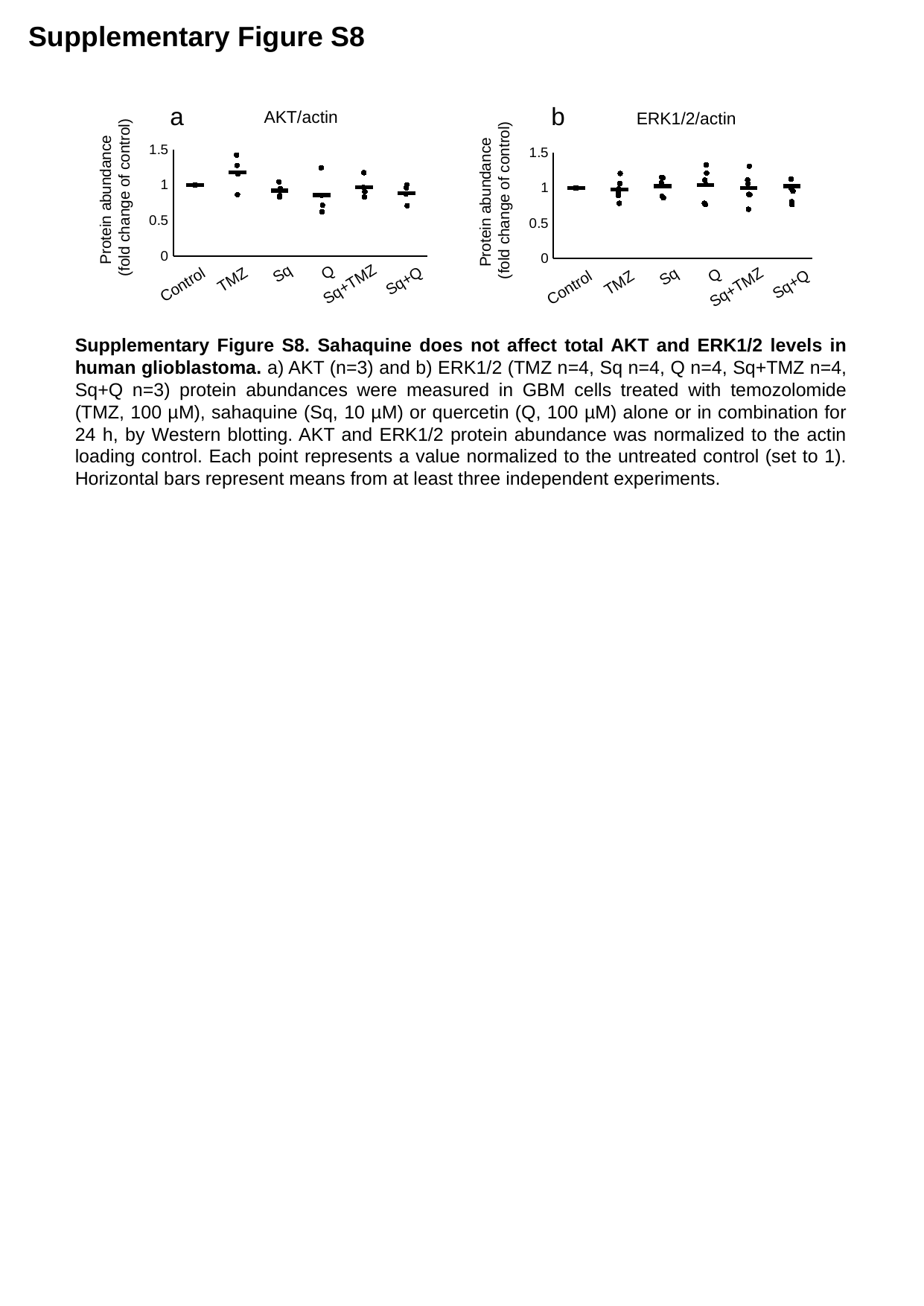

Supplementary Figure S8
a
b
AKT/actin
ERK1/2/actin
### Chart
| Category | Control | TMZ 100 uM | Sahaq 10 μM | Quercetin 100 μM | Sq10 + TMZ 100 uM | Sq 10 + Q100 | | | Control | TMZ 100 uM | Sahaq 10 μM | Quercetin 100 μM | Sq10 + TMZ 100 uM | Sq 10 + Q100 | | |
|---|---|---|---|---|---|---|---|---|---|---|---|---|---|---|---|---|
### Chart
| Category | Control | TMZ 100 uM | Sahaq 10 μM | Quercetin 100 μM | Sq10 + TMZ 100 uM | Sq 10 + Q100 | | | Control | TMZ 100 uM | Sahaq 10 μM | Quercetin 100 μM | Sq10 + TMZ 100 uM | Sq 10 + Q100 | | |
|---|---|---|---|---|---|---|---|---|---|---|---|---|---|---|---|---|Protein abundance
(fold change of control)
Protein abundance
(fold change of control)
Q
Sq
Q
Sq
TMZ
Control
Sq+Q
Sq+TMZ
TMZ
Control
Sq+Q
Sq+TMZ
Supplementary Figure S8. Sahaquine does not affect total AKT and ERK1/2 levels in human glioblastoma. a) AKT (n=3) and b) ERK1/2 (TMZ n=4, Sq n=4, Q n=4, Sq+TMZ n=4, Sq+Q n=3) protein abundances were measured in GBM cells treated with temozolomide (TMZ, 100 µM), sahaquine (Sq, 10 µM) or quercetin (Q, 100 µM) alone or in combination for 24 h, by Western blotting. AKT and ERK1/2 protein abundance was normalized to the actin loading control. Each point represents a value normalized to the untreated control (set to 1). Horizontal bars represent means from at least three independent experiments.

## Slide 13
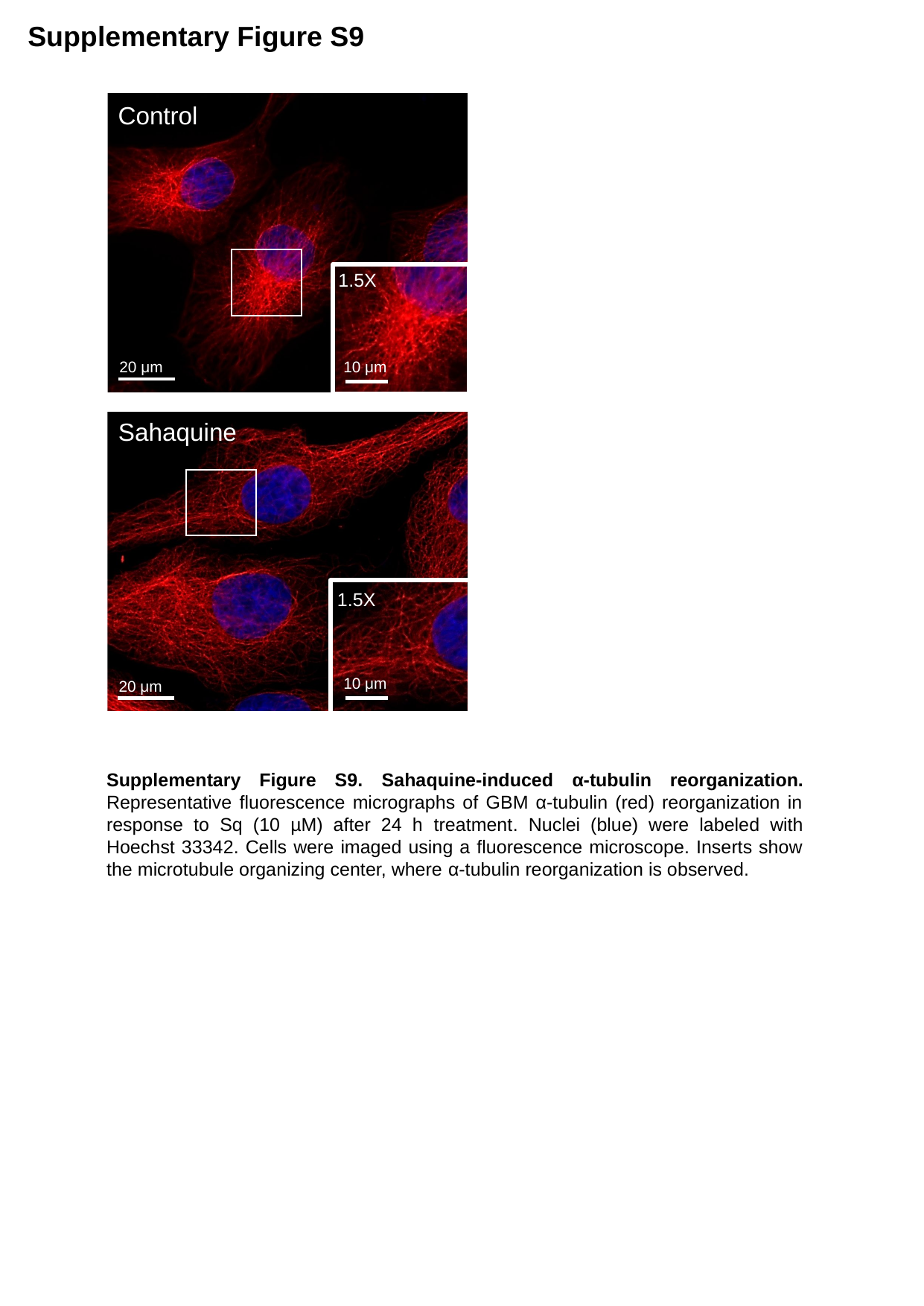

Supplementary Figure S9
Control
1.5X
20 μm
10 μm
Sahaquine
1.5X
10 μm
20 μm
Supplementary Figure S9. Sahaquine-induced α-tubulin reorganization. Representative fluorescence micrographs of GBM α-tubulin (red) reorganization in response to Sq (10 µM) after 24 h treatment. Nuclei (blue) were labeled with Hoechst 33342. Cells were imaged using a fluorescence microscope. Inserts show the microtubule organizing center, where α-tubulin reorganization is observed.

## Slide 14
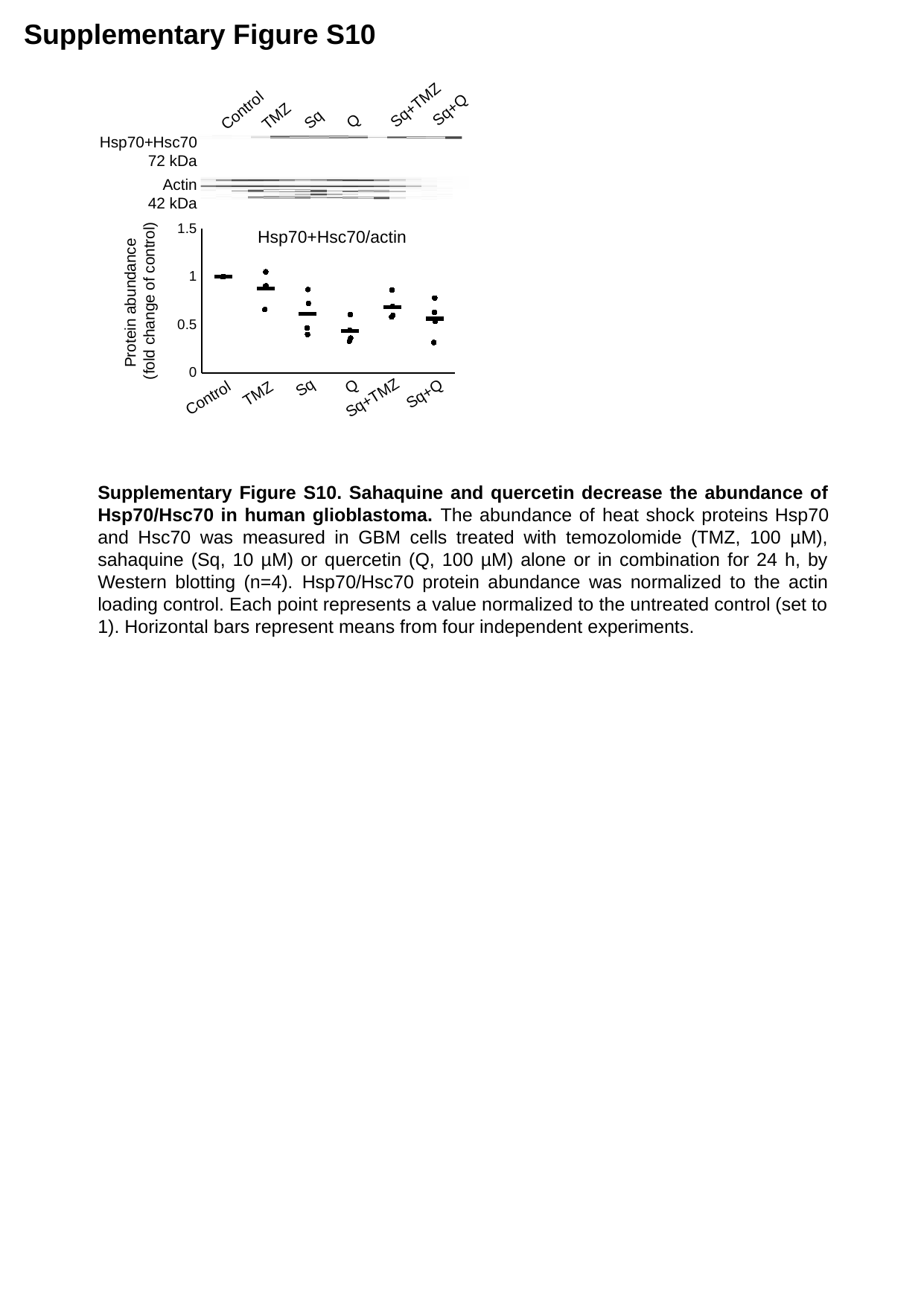

Supplementary Figure S10
Sq+TMZ
Sq+Q
Control
Sq
TMZ
Q
Hsp70+Hsc70
72 kDa
Actin
42 kDa
### Chart
| Category | Control | TMZ 100 uM | Sahaq 10 μM | Quercetin 100 μM | Sq10 + TMZ 100 uM | Sq 10 + Q100 | | | Control | TMZ 100 uM | Sahaq 10 μM | Quercetin 100 μM | Sq10 + TMZ 100 uM | Sq 10 + Q100 | | |
|---|---|---|---|---|---|---|---|---|---|---|---|---|---|---|---|---|Hsp70+Hsc70/actin
Protein abundance
(fold change of control)
Q
Sq
TMZ
Sq+Q
Control
Sq+TMZ
Supplementary Figure S10. Sahaquine and quercetin decrease the abundance of Hsp70/Hsc70 in human glioblastoma. The abundance of heat shock proteins Hsp70 and Hsc70 was measured in GBM cells treated with temozolomide (TMZ, 100 µM), sahaquine (Sq, 10 µM) or quercetin (Q, 100 µM) alone or in combination for 24 h, by Western blotting (n=4). Hsp70/Hsc70 protein abundance was normalized to the actin loading control. Each point represents a value normalized to the untreated control (set to 1). Horizontal bars represent means from four independent experiments.

## Slide 15
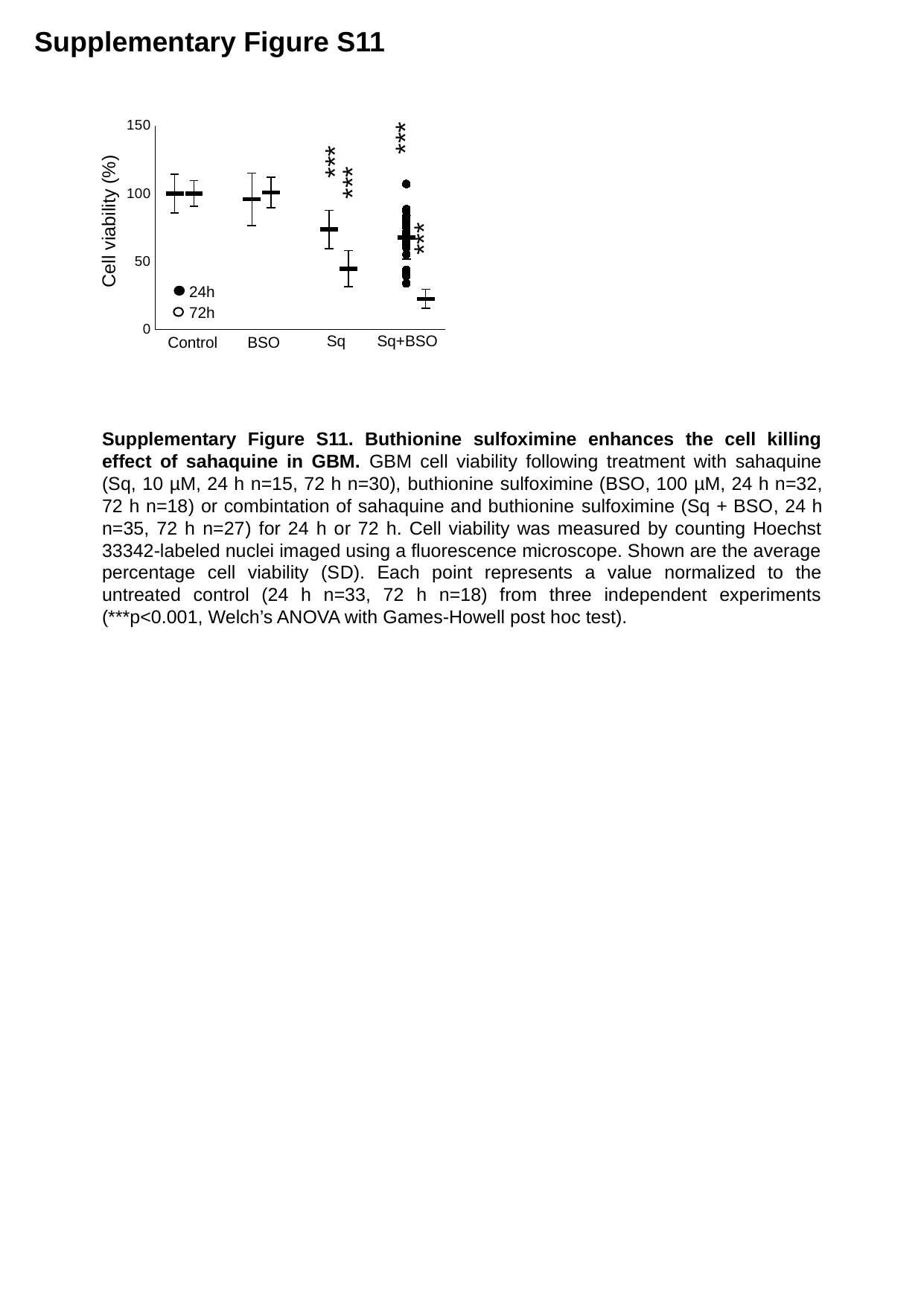

Supplementary Figure S11
### Chart
| Category | Control | BSO | Sq (10uM) | BSO+Sq | Control | BSO | Sq | BSO+Sq | Control | Control | BSO (100uM) | BSO (100uM) | Sq (10uM) | Sq (10uM) | Sq+BSO | Sq+BSO |
|---|---|---|---|---|---|---|---|---|---|---|---|---|---|---|---|---|***
***
***
Cell viability (%)
***
24h
72h
Sq
Sq+BSO
Control
BSO
Supplementary Figure S11. Buthionine sulfoximine enhances the cell killing effect of sahaquine in GBM. GBM cell viability following treatment with sahaquine (Sq, 10 µM, 24 h n=15, 72 h n=30), buthionine sulfoximine (BSO, 100 µM, 24 h n=32, 72 h n=18) or combintation of sahaquine and buthionine sulfoximine (Sq + BSO, 24 h n=35, 72 h n=27) for 24 h or 72 h. Cell viability was measured by counting Hoechst 33342-labeled nuclei imaged using a fluorescence microscope. Shown are the average percentage cell viability (SD). Each point represents a value normalized to the untreated control (24 h n=33, 72 h n=18) from three independent experiments (***p<0.001, Welch’s ANOVA with Games-Howell post hoc test).
